# A Simple Method for Comparing Complex Models: Bayesian Model Comparison for Hierarchical Multinomial Processing Tree Models Using Warp-III Bridge Sampling

**DOI:** 10.1007/s11336-018-9648-3

**Published:** 2018-11-27

**Authors:** Quentin F. Gronau, Eric-Jan Wagenmakers, Daniel W. Heck, Dora Matzke

**Affiliations:** 1 University of Amsterdam, Nieuwe Achtergracht 129 B, 1018 WT Amsterdam, The Netherlands; 2 University of Mannheim, Mannheim, Germany

**Keywords:** multinomial processing tree, Bayesian model comparison, Bayes factor, bridge sampling, Warp-III, posterior model probability, Bayesian model averaging

## Abstract

Multinomial processing trees (MPTs) are a popular class of cognitive models for categorical data. Typically, researchers compare several MPTs, each equipped with many parameters, especially when the models are implemented in a hierarchical framework. A Bayesian solution is to compute posterior model probabilities and Bayes factors. Both quantities, however, rely on the marginal likelihood, a high-dimensional integral that cannot be evaluated analytically. In this case study, we show how Warp-III bridge sampling can be used to compute the marginal likelihood for hierarchical MPTs. We illustrate the procedure with two published data sets and demonstrate how Warp-III facilitates Bayesian model averaging.

**Electronic supplementary material:**

The online version of this article (10.1007/s11336-018-9648-3) contains supplementary material, which is available to authorized users.

Multinomial processing trees (MPTs; e.g., Riefer & Batchelder, 1988) are substantively motivated stochastic models for the analysis of categorical data. MPTs allow researchers to test theories about cognitive architecture by formalizing qualitatively different cognitive processes that underlie performance in an experimental paradigm. MPTs are popular in various areas of psychology and have been applied, for instance, in research on memory, perception, logical reasoning, and attitudes (for reviews, see Batchelder & Riefer, 1999; Erdfelder et al., 2009; Hütter & Klauer, 2016). MPTs are related to tree-based item response theory models as presented, for instance, in Böckenholt (2012a, b); Culpepper (2014), and De Boeck and Partchev (2012).^1^

Traditionally, parameter estimation in MPTs has relied on maximum likelihood methods for aggregated data (Hu & Batchelder, 1994; Singmann & Kellen, 2013). Recently, however, MPT modelers have become increasingly interested in using Bayesian hierarchical methods to examine individual differences in model parameters (Klauer, 2010; Matzke, Dolan, Batchelder, & Wagenmakers, 2015; Smith & Batchelder, 2010). Bayesian hierarchical modeling allows researchers to simultaneously account for the differences and similarities between participants and typically provides more accurate statistical inference than the analysis of aggregated data, especially in situations with moderate between-subject variability and scarce participant-level data (e.g., Gelman & Hill, 2007).

In typical applications, MPT modelers are interested in comparing a limited set of models. The models can be nested, which is the case when testing parameter constraints (e.g., Batchelder & Riefer, 1990; Singmann, Kellen, & Klauer, 2013), or non-nested, which is the case when comparing structurally different models (e.g., Fazio, Brashier, Payne, & Marsh, 2015; Kellen, Singmann, & Klauer, 2014). A wide range of model comparison and assessment methods exist both in the frequentist and Bayesian framework, each with its own goals and operating characteristics, such as Pearson’s $\chi ^2$ test, the likelihood ratio test, information criteria such as AIC (Akaike, 1973), BIC (Schwarz, 1978), DIC (Spiegelhalter, Best, Carlin, & van der Linde, 2002), and WAIC (Watanabe, 2010), leave-one-out cross-validation (Vehtari, Gelman, & Gabry, 2017), and posterior predictive checks (Gelman, 2013; Meng, 1994; Robins, van der Vaart, & Ventura, 2000). Furthermore, a range of powerful methods exist for analyzing multinomial data in particular (e.g., Bishop, Fienberg, & Holland, 1975; Maydeu-Olivares & Joe, 2005). The goal of this case study is to enrich the model comparison toolkit of MPT modelers by illustrating—with examples from the literature—a computationally feasible approach to model comparison in hierarchical MPTs based on Bayes factors and posterior model probabilities.^2^ Furthermore, the proposed approach also enables Bayesian model averaging which we advocate as a principled way of testing parameter constraints while fully taking into account model uncertainty.

Suppose one is interested in comparing a discrete set of *M* models denoted as $\mathcal {M}_1, \mathcal {M}_2, \ldots , \mathcal {M}_M$ with corresponding prior model probabilities $p(\mathcal {M}_1), p(\mathcal {M}_2), \ldots , p(\mathcal {M}_M)$, which satisfy the constraints $p(\mathcal {M}_i) \ge 0 \; \; \forall i \in \{1, 2, \ldots , M\}$ and $\sum _{i=1}^{M} p(\mathcal {M}_i) = 1$. The posterior model probability of $\mathcal {M}_i$ is then obtained using Bayes’ rule:

1 $$\begin{aligned} \underbrace{p(\mathcal {M}_i \mid \text {data})}_{\text {posterior model probability}} = \underbrace{\frac{p(\text {data} \mid \mathcal {M}_i)}{\sum _{j=1}^{M} p(\text {data} \mid \mathcal {M}_j) \, p(\mathcal {M}_j)}}_{\text {updating factor}} \;\;\;\;\;\; \times \underbrace{p(\mathcal {M}_i)}_{{\text {prior model probability}}}, \end{aligned}$$

where $p(\text {data} \mid \mathcal {M}_i)$ is the *marginal likelihood* of model $\mathcal {M}_i$.

If model comparison involves assessing the tenability of parameter constraints in a set of nested models, posterior model probabilities can be used to quantify the model-averaged evidence that a parameter is free to vary or should be constrained across different groups or experimental conditions (e.g., Hoeting, Madigan, Raftery, & Volinsky, 1999; Rouder, Morey, Verhagen, Swagman, & Wagenmakers, 2017). If the model comparison involves only two models, $\mathcal {M}_1$ and $\mathcal {M}_2$, it is convenient to consider the odds of one model over the other one. Bayes’ rule yields:

2 $$\begin{aligned} \underbrace{\frac{p(\mathcal {M}_1 \mid \text {data})}{p(\mathcal {M}_2 \mid \text {data})}}_{\text {posterior odds}} = \underbrace{\frac{p(\text {data} \mid \mathcal {M}_1)}{p(\text {data} \mid \mathcal {M}_2)}}_{\text {Bayes factor BF }_{12}} \times \underbrace{\frac{p(\mathcal {M}_1)}{p(\mathcal {M}_2)}}_{\text {prior odds}}. \end{aligned}$$

Equation (2) shows that the change in odds brought about by the data is given by the ratio of the marginal likelihoods of the models, a quantity known as the *Bayes factor* (Etz & Wagenmakers, 2017; Jeffreys, 1961; Kass & Raftery, 1995; Ly, Verhagen, & Wagenmakers, 2016).

Equations (1) and (2) illustrate that the computation of posterior model probabilities and Bayes factors requires the computation of the marginal likelihood of the models. The marginal likelihood is obtained by integrating out the model parameters with respect to the parameters’ prior distribution:

3 $$\begin{aligned} p(\text {data} \mid \mathcal {M}_i) = \int _{\varvec{\Theta }} p(\text {data} \mid \varvec{\theta }, \mathcal {M}_i) p(\varvec{\theta } \mid \mathcal {M}_i) \text {d}\varvec{\theta }. \end{aligned}$$

The marginal likelihood includes a natural penalty for overdue model complexity and implements a form of the principle of parsimony also known as *Occam’s razor* (e.g., Jefferys & Berger, 1992; Myung & Pitt, 1997; Vandekerckhove, Matzke, & Wagenmakers, 2015).^3^ Although conceptually straightforward, in practice it is challenging to compute Bayes factors and posterior model probabilities for hierarchical MPTs because the marginal likelihood features a high-dimensional integral that cannot be solved analytically.

In this case study, we show how Warp-III bridge sampling (Meng & Schilling, 2002; Meng & Wong, 1996, henceforth referred to as Warp-III ) can be used to estimate the marginal likelihood for hierarchical MPTs. Warp-III may be used for nested and, crucially, also non-nested model comparisons, for which simpler methods, such as the Savage–Dickey density ratio (Dickey & Lientz, 1970), cannot be applied. Importantly, Warp-III is not specific to hierarchical MPTs; it may be used to compute the marginal likelihood for a wide range of complex cognitive models. In fact, Warp-III improves upon simpler bridge sampling techniques (e.g., DiCiccio, Kass, Raftery, & Wasserman, 1997, Gronau et al., 2017) by respecting potential skewness in the posterior distribution—a typical consequence of estimating parameters of cognitive models from scarce data (e.g., Ly et al., 2018; Matzke et al., 2015). Due to its accuracy and relatively straightforward implementation, we believe that Warp-III is a promising and timely addition to the Bayesian toolkit of cognitive modelers in general and MPT modelers in particular.

The article is organized as follows. We first introduce the latent-trait approach to hierarchical MPTs. We then demonstrate how Warp-III can be used to estimate the marginal likelihood for latent-trait MPTs. Lastly, we apply the method to two model comparison problems from published studies. The first example focuses on Bayesian model averaging for nested models; the second example focuses on the computation of the Bayes factor for non-nested models.

## Multinomial Processing Trees

Data for MPTs consist of categorical responses^4^ from several participants to a set of items. MPTs are based on the assumption that these responses follow a multinomial distribution. MPTs reparametrize the category probabilities of the multinomial distribution in terms of the model parameters that represent the probabilities of latent cognitive processes (Riefer & Batchelder, 1988).

**Fig. 1:**
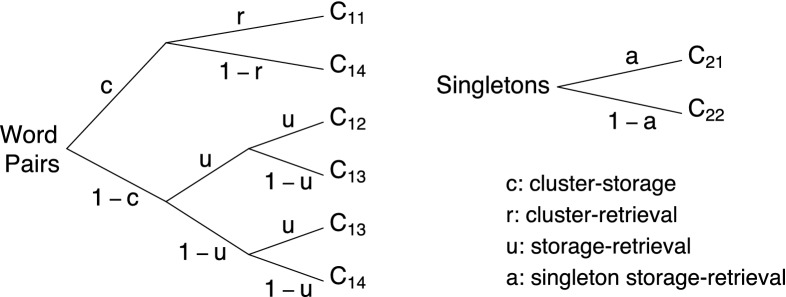
Pair-clustering MPT. Available at https://tinyurl.com/yb7bma4e under CC license https://creativecommons.org/licenses/by/2.0/.

Consider the pair-clustering MPT depicted in Fig. 1. The model was developed for the measurement of the storage and retrieval processes that determine the recall of semantically related word pairs (Batchelder & Riefer, 1980). A typical pair-clustering study involves a free recall memory experiment, where participants are presented with a list of study words in a word-by-word fashion. The study list consists of two types of items: semantically related word pairs such as *knife–fork* and words without a category partner (i.e., singletons), such as *dog*. After the study phase, participants are required to recall as many of the study words as they can. Typically, semantically related word pairs are recalled consecutively as a “pair-cluster.”

The model represents the interplay between the hypothesized latent cognitive processes in a rooted tree structure. The pair-clustering MPT features $K = 2$ independent category systems. Each category system corresponds to a separate multinomial distribution: one for word pairs ($k = 1$) and one for singletons ($k = 2$). The category probabilities in each system are modeled using a separate subtree with a finite number of branches.

Each branch of a subtree corresponds to a specific sequence of processing stages and terminates in one of $L_k$ possible response categories denoted as $C_{kl}$, where $l = 1, \ldots , L_k$ indexes the *l*th of $L_k$ possible responses in subtree *k*. In the pair-clustering MPT, the recall of word pairs is scored into $L_1 = 4$ categories: (1) Both words of the pair are recalled consecutively ($C_{11}$); (2) both words are recalled but not consecutively ($C_{12}$); (3) only one word is recalled ($C_{13}$); (4) no word is recalled ($C_{14}$). The recall of singletons is scored into $L_2 = 2$ response categories: (1) The word is recalled ($C_{21}$); (2) the word is not recalled ($C_{22}$).

The response category probabilities are expressed as a function of the MPT parameters, $\theta _p \in (0, 1) \; \; \forall p \in \{1, 2, \ldots , P\}$, which can be collected in a vector $\varvec{\theta } = (\theta _1, \theta _2, \ldots , \theta _P)$. The pair-clustering MPT features four parameters: $\varvec{\theta } = (c, r, u, a)$. The *cluster-storage* parameter *c* corresponds to the probability that a word pair is stored as a cluster in memory. The *cluster-retrieval* parameter *r* corresponds to the conditional probability that a clustered word pair is retrieved from memory during the test phase. The model assumes that stored and retrieved word clusters are always recalled consecutively. The *storage-retrieval* parameter *u* corresponds to the conditional probability that a member of a word pair is stored and retrieved, given that the word pair was not clustered. The model makes the simplifying assumption that words from unclustered pairs are never recalled consecutively. The *singleton storage-retrieval* parameter *a* corresponds to the probability that a singleton is stored and retrieved. In many applications, researchers impose the constraint that $a = u$.

The response category probabilities are obtained as follows. First, we obtain the probability of each branch that terminates in a given response category. Let $B_{klm}$ denote the *m*th of $M_{kl}$ branches that terminate in response category $C_{kl}$. The probability of branch $B_{klm}$ is obtained by traversing the tree from root to leaf and multiplying the encountered parameters:

4 $$\begin{aligned} \text {Pr}(B_{klm} \mid \varvec{\theta }) = \prod ^P_{p=1}\theta ^{v_{klmp}}_{p} (1-\theta _p)^{w_{klmp}}, \end{aligned}$$

where ${v_{klmp}} \ge 0$ and ${w_{klmp}}\ge 0$ are the number of nodes on branch $B_{klm}$ that are related to parameter $\theta _p$, $p=1, \ldots , P$, and $1-\theta _p$, respectively. Second, we sum the probabilities of the $M_{kl}$ branches that terminate in $C_{kl}$:

5 $$\begin{aligned} \text {Pr}(C_{kl} \mid \varvec{\theta }) = \sum _{m = 1}^{M_{kl}} \text {Pr}(B_{klm} \mid \varvec{\theta }). \end{aligned}$$

For instance, the probability of response category $C_{14}$ is given by $\text {Pr}(C_{14} \mid \varvec{\theta }) = c \; (1 - r) + (1 -c) \; (1 - u)^2$.

The probability of the observed response frequencies across category systems denoted by $\varvec{n} = (n_{11},\ldots , n_{1L_1}, \ldots , n_{K1}, \ldots , n_{KL_K})$, where $n_{kl}$ is the observed response frequency for category $l = 1,\ldots , L_k$ in category system (subtree) $k = 1,\ldots , K$, is given by a product-multinomial distribution:

6 $$\begin{aligned} \text {Pr}(\varvec{N} = \varvec{n} \mid \varvec{\theta }) = \prod ^K_{k=1} \left\{ {\frac{J_k!}{n_{k1}! \times n_{k2}! \times ... \times n_{kL_k}!}} \prod ^{L_k}_{l=1}\left[ \text {Pr}(C_{kl} \mid \varvec{\theta })\right] ^{n_{kl}}\right\} , \end{aligned}$$

where $J_k$ denotes the number of items in category system *k* (see also Klauer, 2010; Matzke et al., 2015).

### Bayesian Hierarchical MPTs: The Latent-Trait Approach

Bayesian hierarchical approaches explicitly model heterogeneity in participants by introducing a group-level distribution from which the participant-level parameters are drawn (e.g., Gelman & Hill, 2007; Gill, 2002; Lee, 2011; Lee & Wagenmakers, 2013; Rouder & Lu, 2005).^5^ Here we focus on Klauer’s (2010) latent-trait approach that relies on a multivariate normal group-level distribution to describe the between-subject variability and the correlations between the participant-level parameters.

To model participant heterogeneity, observed responses are aggregated over items, but not over participants, resulting in a vector of category frequencies for each participant *i*: $\varvec{n}_i$, $i = 1, 2, \ldots , I$, where *I* is the total number of participants. Each participant obtains a participant-specific parameter vector $\varvec{\theta }_i$ of length *P*.

The latent-trait approach assumes that the probit-transformed participant-level parameter vectors $\varvec{\theta }_{i}^{'} = \Phi ^{-1}(\varvec{\theta }_{i})$ follow a *P*-dimensional multivariate normal distribution with mean vector $\varvec{\mu }$ and covariance matrix $\varvec{\Sigma }$: $\varvec{\theta }_{i}^{'} \sim \mathcal {N}_P(\varvec{\mu }, \varvec{\Sigma })$. The probit transformation $\Phi ^{-1}(\varvec{\theta }_i)$ is defined component-wise, where $\Phi ^{-1}(\cdot )$ corresponds to the inverse of the cumulative distribution function of the normal distribution. Priors are assigned to $\varvec{\mu }$ and $\varvec{\Sigma }$. We follow earlier implementations of the latent-trait approach and assign independent standard normal distributions to the *P* components of $\varvec{\mu }$ (Heck, Arnold, & Arnold, 2018a; Matzke et al., 2015). This choice corresponds to uniform priors on the probability scale for the grand means. For the covariance matrix $\varvec{\Sigma }$, a convenient prior choice would be an inverse Wishart prior with degrees of freedom $\nu = P + 1$ and identity scale matrix. This setting leads to uniform priors on the correlation parameters; however, this choice is constraining on the standard deviation parameters. Although changing the degrees of freedom $\nu$ affords more flexibility for modeling the standard deviations, it comes at the cost of constraining the prior on the correlation parameters (Gelman & Hill, 2007).

This dilemma can be circumvented by using a scaled inverse Wishart prior as introduced by Gelman and Hill (2007) and proposed in the context of hierarchical MPT modeling by Klauer (2010). Compared to a regular inverse Wishart prior, the scaled version has the advantage that it allows one to model the standard deviations more flexibly while retaining the desirable uniform prior on the correlation parameters. The scaled inverse Wishart prior is based on the following decomposition of the covariance matrix $\varvec{\Sigma }$:

7 $$\begin{aligned} \varvec{\Sigma } = \text {Diag}(\varvec{\xi }) \, \varvec{Q} \, \text {Diag}(\varvec{\xi }), \end{aligned}$$

where $\varvec{\xi }$ is a vector of *P* scaling parameters and $\varvec{Q}$ corresponds to the $P \times P$ unscaled covariance matrix. The scaled inverse Wishart prior is obtained by placing a regular inverse Wishart prior on the unscaled covariance matrix $\varvec{Q}$ and a suitable prior on the vector of scaling parameters $\varvec{\xi }$.

We follow Klauer (2010) and assign $\varvec{Q}$ an inverse Wishart prior with degrees of freedom $\nu = P + 1$ and scale matrix $\varvec{I}_P$ (i.e., $P \times P$ identity matrix). For the *P* components of $\varvec{\xi }$, we follow Heck et al. (2018a) and use independent uniform priors that range from zero to ten. These choices correspond to relatively diffuse priors for the standard deviations of the random effects on the probit scale and uniform priors for the correlations between the random effects.

Note that these prior distributions have been proposed in a context of parameter estimation, where the exact choice of the prior is irrelevant as long as sufficiently informative data are available. In contrast, in the context of model comparison, the priors have an important and lasting effect: As shown in Eq. (3), the marginal likelihood is obtained by taking a weighted average of the probability of the data across all possible parameter settings where the weights correspond to the parameters’ prior density. We argue that the standard normal and uniform priors for the grand means and the correlations, respectively, provide a reasonable default setting also from the perspective of model comparison. The choice of the prior for $\varvec{\xi }$ is less straightforward. We report the results corresponding to the default setting of the recently developed MPT software package TreeBUGS (Heck et al., 2018a), but we probed the robustness of our conclusions with a sensitivity analysis using $\xi _p \sim \text {Uniform}(0, \xi _{\text {max}}) \, \forall p \in \{1, 2, \ldots , P\}$, with $\xi _{\text {max}} = 2$ instead of $\xi _{\text {max}} = 10$, a prior that was chosen based on the implied group-level distributions on the probability scale. As the conclusions were unaffected by the choice of the upper bound, the results of the sensitivity analysis are mentioned only briefly and are presented in more detail in Supplemental Materials available at https://osf.io/rycg6/.

Under these prior settings, the probit-transformed participant-level MPT parameter vectors can be written as:

8 $$\begin{aligned} \varvec{\theta }_{i}^{'} = \varvec{\mu } + \varvec{\xi } \odot \varvec{\omega }_i, \end{aligned}$$

where $\varvec{\omega }_i$ is the *P*-dimensional vector with the unscaled random effects for participant *i* and $\odot$ denotes the Hadamard product (i.e., entry-wise multiplication, e.g., Liu & Trenkler, 2008). The unscaled random effects are drawn from a *P*-dimensional zero-centered multivariate normal distribution with covariance matrix $\varvec{Q}$: $\varvec{\omega }_i \sim \mathcal {N}_P(\varvec{0},\varvec{Q})$.

Note that the model is overparameterized: $\varvec{\xi }$ and $\varvec{Q}$ cannot be interpreted separately. Similarly, the unscaled random effects $\varvec{\omega }_i$ cannot be interpreted on their own but need to be combined with the scaling parameter vector $\varvec{\xi }$ to form the random effects of interest. The scaling parameters $\varvec{\xi }$, the unscaled covariance matrix $\varvec{Q}$, and the unscaled random effects $\varvec{\omega }_i$ are not of interest in themselves and are simply an artifact of using a flexible scaled inverse Wishart prior on $\varvec{\Sigma }$: The parameters of interest are $\varvec{\theta }_{i}^{'}$, $\varvec{\mu }$, and $\varvec{\Sigma }$. Therefore, the scaled inverse Wishart prior can be regarded as a form of parameter expansion (e.g., Gelman & Hill, 2007) which has been reported to speed up convergence when fitting the model using Markov chain Monte Carlo sampling (MCMC; e.g., Gamerman & Lopes, 2006).

The reader is referred to Klauer (2010) and Matzke et al. (2015) for a more detailed description of the latent-trait approach. Parameter estimation may proceed using MCMC sampling implemented in standard Bayesian statistical software such as JAGS (Plummer, 2003) or Stan (Stan Development Team, 2016).

### Computing the Marginal Likelihood

The marginal likelihood for latent-trait MPTs is given by:^6^

9 $$\begin{aligned} \text {Pr}(\varvec{N} = \varvec{n}) =&\int ... \int \prod _{i = 1}^{I} \left[ \overbrace{\text {Pr}(\varvec{N}_i = \varvec{n}_i \mid \varvec{\mu }, \varvec{\xi }, \varvec{\omega }_i)}^{\text {individual-level}} \overbrace{p(\varvec{\omega }_i \mid \varvec{Q})}^{\text {group-level}} \right] \overbrace{p(\varvec{Q})p(\varvec{\mu }) p(\varvec{\xi })}^{\text {priors}} \text {d}\varvec{Q} \text {d}\varvec{\mu } \text {d}\varvec{\xi } \text {d}\varvec{\omega }_1 ... \text {d}\varvec{\omega }_I \nonumber \\ =&{\int } ... {\int } \prod _{i = 1}^{I} \Bigg [\underbrace{\prod ^K_{k=1} \Bigg \{{\frac{J_k!}{n_{ik1}! \times n_{ik2}! \times ... \times n_{ikL_k}!}} \prod ^{L_k}_{l=1}\left[ \text {Pr}(C_{kl} \mid \varvec{\mu }, \varvec{\xi }, \varvec{\omega }_i)\right] ^{n_{ikl}}\Bigg \}}_{\text {Pr}(\varvec{N}_i = \varvec{n}_i \mid \varvec{\mu }, \varvec{\xi }, \varvec{\omega }_i)} \nonumber \\&\quad \times \underbrace{\left( 2\pi \right) ^{-\frac{P}{2}} \left| \varvec{Q}\right| ^{-\frac{1}{2}} \exp \bigg \{-\frac{1}{2} \varvec{\omega }_i^\top \varvec{Q}^{-1}\varvec{\omega }_i\bigg \}}_{p(\varvec{\omega }_i \mid \varvec{Q})}\Bigg ] \nonumber \\&\quad \times \underbrace{\frac{1}{2^{\frac{\nu P}{2}}\Gamma _P(\frac{\nu }{2})} \left| \varvec{Q}\right| ^{-\frac{\nu +P+1}{2}}\exp \left\{ -\frac{1}{2}{\text {tr}}\left( \varvec{Q}^{-1}\right) \right\} }_{p(\varvec{Q})} \nonumber \\&\quad \times \underbrace{\left( 2\pi \right) ^{-\frac{P}{2}} \exp \bigg \{-\frac{1}{2} \varvec{\mu }^\top \varvec{\mu }\bigg \}}_{p(\varvec{\mu })} \underbrace{\left( \xi _{\text {max}}\right) ^{-P}}_{p(\varvec{\xi })} \text {d}\varvec{Q} \text {d}\varvec{\mu } \text {d}\varvec{\xi } \text {d}\varvec{\omega }_1 ... \text {d}\varvec{\omega }_I, \end{aligned}$$

where $\Gamma _{P}(a)=\pi ^{{P(P-1)/4}}\prod _{{j=1}}^{P}\Gamma \left( a+\frac{1-j}{2}\right)$ and $\Gamma (z)=\int _{0}^{\infty }x^{z-1}e^{-x}\,\text {d}x$ are the multivariate and regular gamma function, respectively. In this parametrization, we do not need to explicitly integrate out the participant-level parameter vectors $\varvec{\theta }_i$ since they are functions of $\varvec{\mu }$, $\varvec{\xi }$, and $\varvec{\omega }_i$ (see Eq. (8)).

We exploit the fact that the covariance matrix $\varvec{Q}$ in Eq. (9) can be integrated out in closed form (see also, Overstall & Forster, 2010); a detailed derivation is provided in Supplemental Materials. The marginal likelihood is then given by:

10 $$\begin{aligned} \text {Pr}(\varvec{N} = \varvec{n})= & {} {\int } ... {\int } \prod _{i = 1}^{I} \left[ \prod ^K_{k=1} \Bigg \{{\frac{J_k!}{n_{ik1}! \times n_{ik2}! \times ... \times n_{ikL_k}!}} \prod ^{L_k}_{l=1}\left[ \text {Pr}(C_{kl} \mid \varvec{\mu }, \varvec{\xi }, \varvec{\omega }_i)\right] ^{n_{ikl}}\Bigg \}\right] \nonumber \\&\quad \times \frac{\Gamma _P(\frac{\nu + I}{2})}{\Gamma _P(\frac{\nu }{2})} \frac{\pi ^{-\frac{I P}{2}}}{\left| { \varvec{\Omega }^\top \varvec{\Omega } + {\varvec{I}_P}}\right| ^{\frac{\nu + I}{2}}} \times \left( 2\pi \right) ^{-\frac{P}{2}} \exp \bigg \{-\frac{1}{2} \varvec{\mu }^\top \varvec{\mu }\bigg \}\nonumber \\&\quad \times \left( \xi _{\text {max}}\right) ^{-P} \text {d}\varvec{\mu } \text {d}\varvec{\xi } \text {d}\varvec{\omega }_1 ... \text {d}\varvec{\omega }_I, \end{aligned}$$

where $\varvec{\Omega }$ is an $I \times P$ matrix of the *P*-dimensional random effects vectors $\varvec{\omega }_i$ of the *I* participants. Even after integrating out $\varvec{Q}$, the expression for the marginal likelihood is still a high-dimensional integral (i.e., $P(I + 2)$ dimensions); the challenge is to find a method which yields accurate estimates of this integral.

## Warp-III Bridge Sampling for MPTs

We propose to use Warp-III bridge sampling (Meng & Schilling, 2002; Meng & Wong, 1996; Overstall, 2010), an advanced version of bridge sampling, to evaluate the high-dimensional integral in Eq. (10). Bridge sampling is a general method for estimating normalizing constants^7^, a problem that is not only encountered in Bayesian inference, but also in likelihood-based approaches (Gelman & Meng, 1998). We first outline the basic principles of bridge sampling and then present the details of the advanced Warp-III method. The reader is referred to the recent tutorial by Gronau et al. (2017) for a detailed explanation of the general bridge sampling approach.

Let $\varvec{\zeta } = (\varvec{\mu }, \varvec{\xi }, \varvec{\omega }_1, \ldots , \varvec{\omega }_I)$ be the vector of quantities that must be integrated out to obtain the marginal likelihood, so that

11 $$\begin{aligned} \begin{aligned} \text {Pr}(\varvec{N} = \varvec{n}) =&\int \text {Pr}(\varvec{N} = \varvec{n} \mid \varvec{\zeta }) \, p(\varvec{\zeta }) \text {d}\varvec{\zeta }. \end{aligned} \end{aligned}$$

General bridge sampling is based on the following identity:

12 $$\begin{aligned} 1 = \frac{ {\int } \overbrace{h(\varvec{\zeta })}^{\text {bridge function}} \, p(\varvec{\zeta } \mid \varvec{N} = \varvec{n}) \, \overbrace{g(\varvec{\zeta })}^{\text {proposal distribution}} \text {d}\varvec{\zeta }}{{\int } h(\varvec{\zeta }) \underbrace{p(\varvec{\zeta } \mid \varvec{N} = \varvec{n})}_{\text {posterior distribution}} g(\varvec{\zeta }) \text {d}\varvec{\zeta }}, \end{aligned}$$

where $p(\varvec{\zeta } \mid \varvec{N} = \varvec{n})$ is the posterior distribution of $\varvec{\zeta }$, $g(\varvec{\zeta })$ is the probability density function of a proposal distribution, and $h(\varvec{\zeta })$ is a function such that $0< \left| \int h(\varvec{\zeta }) \, p(\varvec{\zeta } \mid \varvec{N} = \varvec{n}) \, g(\varvec{\zeta }) \text {d}\varvec{\zeta }\right| < \infty$. It follows from Eq. (12) that

13 $$\begin{aligned} \text {Pr}(\varvec{N} = \varvec{n}) = \frac{{\int } h(\varvec{\zeta }) \, \text {Pr}(\varvec{N} = \varvec{n} \mid \varvec{\zeta }) \, p(\varvec{\zeta }) \, g(\varvec{\zeta }) \text {d}\varvec{\zeta }}{{\int } h(\varvec{\zeta }) \, g(\varvec{\zeta }) \, p(\varvec{\zeta } \mid \varvec{N} = \varvec{n}) \text {d}\varvec{\zeta }} = \frac{\mathbb {E}_{g(\varvec{\zeta })} \left[ h(\varvec{\zeta }) \, \text {Pr}(\varvec{N} = \varvec{n} \mid \varvec{\zeta }) \, p(\varvec{\zeta })\right] }{\mathbb {E}_{p(\varvec{\zeta } \mid \varvec{N} = \varvec{n})} \left[ h(\varvec{\zeta }) \, g(\varvec{\zeta })\right] }. \end{aligned}$$

The bridge sampling estimate of the marginal likelihood is then obtained by sampling from $g(\varvec{\zeta })$ and $p(\varvec{\zeta } \mid \varvec{N} = \varvec{n})$ and then using Monte Carlo approximations to estimate the expected values.

The optimal choice of $h(\varvec{\zeta })$, one that minimizes the relative mean-squared error of the estimator, is given by:

14 $$\begin{aligned} h_o(\varvec{\zeta }) \propto \left[ s_1 \, \text {Pr}(\varvec{N} = \varvec{n} \mid \varvec{\zeta }) \, p(\varvec{\zeta }) + s_2 \, \text {Pr}(\varvec{N} = \varvec{n}) \, g(\varvec{\zeta }) \right] ^{-1}, \end{aligned}$$

where $s_i = \frac{D_i}{D_1 + D_2}, \; i \in \{1,2\}$, where $D_1$ and $D_2$ denote the number of draws from $p(\varvec{\zeta } \mid \varvec{N} = \varvec{n})$ and $g(\varvec{\zeta })$, respectively, used to approximate the expected values (Meng & Wong, 1996). We set $D_1 = D_2$. Note that $h_o$ is only optimal if the draws from the posterior distribution are independent which is not the case with MCMC procedures. To account for this fact, we replace $D_1$ in defining the weights $s_1$ and $s_2$ by the effective sample size obtained using the codaR package (Plummer, Best, Cowles, & Vines, 2006).^8^ As $h_o(\varvec{\zeta })$ depends on $\text {Pr}(\varvec{N} = \varvec{n})$, the very quantity we want to estimate, we follow Meng and Wong (1996) and use an iterative scheme to update an initial guess of the marginal likelihood until convergence:^9^

15 $$\begin{aligned} \hat{\text {Pr}}(\varvec{N} = \varvec{n})^{(t+1)} = \frac{\frac{1}{D_2}\sum \limits _{r = 1}^{D_2}\frac{l_{2, r}}{s_1 l_{2, r} + s_2 \hat{\text {Pr}}(\varvec{N} = \varvec{n})^{(t)}}}{\frac{1}{D_1}\sum \limits _{j = 1}^{D_1}\frac{1}{s_1 l_{1, j} + s_2 \hat{\text {Pr}}(\varvec{N} = \varvec{n})^{(t)}}}, \end{aligned}$$

where $l_{1,j} = \frac{\text {Pr}(\varvec{N} = \varvec{n} \mid \varvec{\zeta ^*}_j) \, p(\varvec{\zeta ^*}_j)}{g(\varvec{\zeta ^*}_j)}$, $l_{2,r} = \frac{\text {Pr}(\varvec{N} = \varvec{n} \mid \varvec{\tilde{\zeta }}_r) \, p(\varvec{\tilde{\zeta }}_r)}{g(\varvec{\tilde{\zeta }}_r)}$, $\{\varvec{\zeta ^*}_1, \ldots , \varvec{\zeta ^*}_{D_1}\}$ are $D_1$ draws from $p(\varvec{\zeta } \mid \varvec{N} = \varvec{n})$, and $\{\varvec{\tilde{\zeta }}_1, \ldots , \varvec{\tilde{\zeta }}_{D_2}\}$ are $D_2$ draws from $g(\varvec{\zeta })$.

A remaining question is how to choose $g(\varvec{\zeta })$. The precision of the bridge sampling estimator is governed by the number of samples from $g(\varvec{\zeta })$ and the overlap between $g(\varvec{\zeta })$ and $p(\varvec{\zeta } \mid \varvec{N} = \varvec{n})$ (Meng & Wong, 1996). Therefore, $g(\varvec{\zeta })$ should closely resemble the posterior distribution. For instance, we may choose a multivariate normal distribution for *g* with mean vector and covariance matrix that match the corresponding quantities of the posterior samples. Although the multivariate normal approach works well in many applications (e.g., Gronau et al., 2017; Overstall & Forster, 2010), it can be inefficient when the posterior distribution is skewed.

Warp-III improves upon the multivariate normal bridge sampling approach by matching, not only the first two, but also the third moment (i.e., skewness) of *g* and the posterior distribution. Consequently, in case there is no skewness, Warp-III results in estimates with the same precision as the ones from the simpler multivariate normal approach. However, crucially, in the presence of skewness, Warp-III is able to match *g* and the posterior distribution more closely which results in a higher precision of the marginal likelihood estimates compared to the simpler approach. How much of an improvement Warp-III is over the simpler multivariate normal approach may depend on the particular example at hand.

**Fig. 2:**
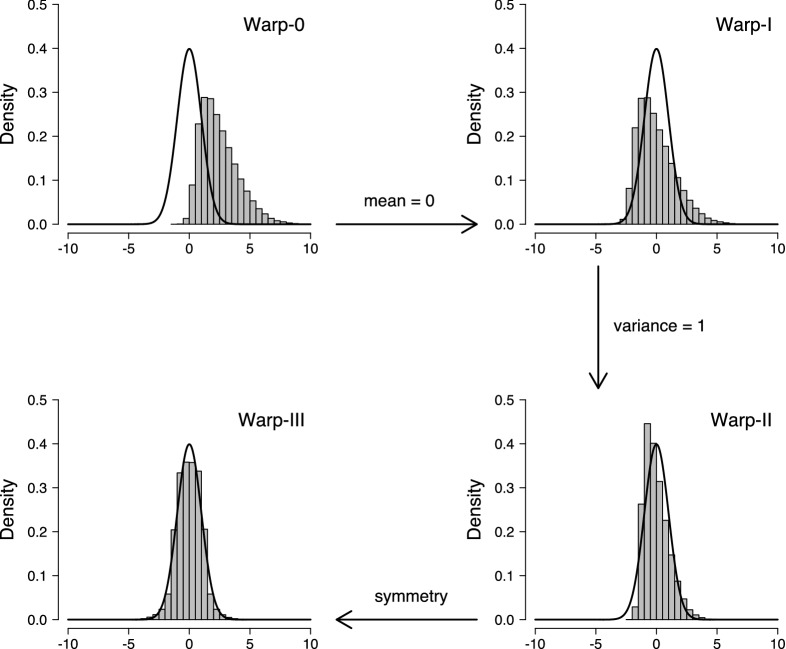
Matching the proposal and posterior distribution with warping. Histograms show the posterior distribution; density lines show the standard normal proposal distribution. Available at https://tinyurl.com/y7owvsz3 under CC license https://creativecommons.org/licenses/by/2.0/.

In Warp-III, *g* is fixed to a multivariate standard normal distribution. The posterior distribution is then manipulated—“warped”—so that its mean vector, covariance matrix, and skew match *g*. Crucially, the warped posterior distribution retains the normalizing constant of the posterior distribution. Figure 2 illustrates the rationale of the Warp-III transformation for the univariate case. The histogram in the upper-left panel shows hypothetical “unbounded” posterior samples that can range across the entire real line; the solid line shows the standard normal proposal distribution *g*. The overlap between the two distributions is clearly suboptimal. Bridge sampling applied to these two distributions can be thought of as “Warp-0” because the posterior distribution is not modified. The upper-right panel illustrates “Warp-I”: Subtracting the mean of the posterior samples from all posterior samples matches the first moment of the distributions. The lower-right panel illustrates “Warp-II”: Dividing the zero-centered posterior samples by their standard deviation matches the first two moments of the distributions. This approach is practically equivalent to the multivariate normal bridge sampling approach described above. Lastly, the lower-left panel illustrates Warp-III: Randomly assigning a minus sign to the standardized posterior samples matches also the third moment of the distributions.

Warp-III assumes that all components of the parameter vector can range across the entire real line. In the context of latent-trait MPTs, this assumption is not fulfilled since $\xi _p \in (0, \xi _{\text {max}}) \; \forall p \in \{1, \ldots , P\}$. We therefore transform $\varvec{\xi }$ so that $\varvec{\xi }_{\text {trans}} = \Phi ^{-1}\left( \frac{\varvec{\xi }}{\xi _{\text {max}}}\right)$ with Jacobian $\left( \xi _{\text {max}}\right) ^{P} \mathcal {N}_P(\varvec{\xi }_{\text {trans}}; \varvec{0}, \varvec{I}_P)$, where $\mathcal {N}_P(\varvec{x}; \varvec{y}, \varvec{Z})$ denotes the probability density function of a *P*-dimensional normal distribution with mean vector $\varvec{y}$ and covariance matrix $\varvec{Z}$ which is evaluated for the vector $\varvec{x}$.^10^ Let $\varvec{\psi } = (\varvec{\mu }, \varvec{\xi }_{\text {trans}} , \varvec{\omega }_1, \ldots , \varvec{\omega }_I)$ denote the resulting parameter vector where all components are on the real line.

Warp-III is then based on applying the following stochastic transformation to $\varvec{\psi }$:

16 $$\begin{aligned} \varvec{\eta } = \underbrace{b}_{\textstyle \text {symmetry}} \times \underbrace{\varvec{R}^{-1}}_{\textstyle \text {covariance } \varvec{I}} \times \;\; \underbrace{(\varvec{\psi } - \varvec{v})}_{\textstyle \text {mean } \varvec{0}}, \end{aligned}$$

where $b \sim \text {Bernoulli}(0.5)$ on $\{-1, 1\}$ and $\varvec{v}$ corresponds to the expected value of $\varvec{\psi }$ (i.e., the mean vector). The matrix $\varvec{R}$ is obtained via the Cholesky decomposition of the covariance matrix of $\varvec{\psi }$, denoted as $\varvec{S}$, thus, $\varvec{S} = \varvec{R} \varvec{R}^\top$. In practice, $\varvec{v}$ and $\varvec{S}$ are unknown and must be approximated using the posterior samples. Note that Eq. (16) simply generalizes the intuition illustrated in Fig. 2 for the univariate case to the general case with multiple parameters.

Due to the Bernoulli random variable *b*, the warped posterior density has the form of a mixture density (see also Overstall, 2010, p. 70):

17 $$\begin{aligned} \begin{aligned} p_{\varvec{\eta }}(\varvec{\eta } \mid \varvec{N} = \varvec{n})&= \frac{\left| \varvec{R}\right| }{2} \left[ \frac{\tilde{p}_{\varvec{\psi }}(\varvec{v} - \varvec{R}\varvec{\eta } \mid \varvec{N} = \varvec{n})}{\text {Pr}(\varvec{N} = \varvec{n})} + \frac{\tilde{p}_{\varvec{\psi }}(\varvec{v} + \varvec{R}\varvec{\eta } \mid \varvec{N} = \varvec{n})}{\text {Pr}(\varvec{N} = \varvec{n})}\right] \\&= \frac{\tilde{p}_{\varvec{\eta }}(\varvec{\eta } \mid \varvec{N} = \varvec{n})}{\text {Pr}(\varvec{N} = \varvec{n})}, \end{aligned} \end{aligned}$$

where $\tilde{p}_{\varvec{\eta }}(\varvec{\eta } \mid \varvec{N} = \varvec{n}) = \frac{\left| \varvec{R}\right| }{2} \left[ \tilde{p}_{\varvec{\psi }}(\varvec{v} - \varvec{R}\varvec{\eta } \mid \varvec{N} = \varvec{n}) + \tilde{p}_{\varvec{\psi }}(\varvec{v} + \varvec{R}\varvec{\eta } \mid \varvec{N} = \varvec{n})\right]$ denotes the un-normalized warped posterior distribution and $\tilde{p}_{\varvec{\psi }}(\cdot \mid \varvec{N} = \varvec{n})$ denotes the un-normalized posterior distribution that has been transformed to the real line (but not warped). This proves that the warped posterior distribution retains the normalizing constant of the original posterior distribution.

The Warp-III estimator of the marginal likelihood is then derived by using the warped posterior distribution $p_{\varvec{\eta }}(\varvec{\eta } \mid \varvec{N} = \varvec{n})$ instead of $p(\varvec{\zeta } \mid \varvec{N} = \varvec{n})$ in Eq. (12). Equation (13) shows that this results in a ratio of two expected values, where the numerator is an expected value with respect to the multivariate standard normal proposal distribution $g(\varvec{\eta })$ and the denominator is an expected value with respect to the warped posterior distribution $p_{\varvec{\eta }}(\varvec{\eta } \mid \varvec{N} = \varvec{n})$. Hence, we could obtain an estimate of the marginal likelihood by first warping the posterior samples using Eq. (16), then sampling from the proposal distribution, and applying the iterative updating scheme in Eq. (15).

However, in line with the literature (e.g., Sinharay & Stern, 2005), we rewrite the expected value in the denominator of Eq. (13) in terms of the unbounded posterior samples that are transformed to the real line but are not warped; a derivation is provided in Supplemental Materials. The estimate of the marginal likelihood is then obtained by applying the iterative scheme in Eq. (15) using:

18 $$\begin{aligned} l_{1,j} = \frac{\frac{\left| \varvec{R}\right| }{2} \left[ \tilde{p}_{\varvec{\psi }}(2\varvec{v} - \varvec{\psi ^*}_j \mid \varvec{N} = \varvec{n}) + \tilde{p}_{\varvec{\psi }}(\varvec{\psi ^*}_j \mid \varvec{N} = \varvec{n})\right] }{g\left( \varvec{R}^{-1}\left( \varvec{\psi ^*}_j - \varvec{v}\right) \right) }, \end{aligned}$$

and

19 $$\begin{aligned} l_{2,r} =\frac{\frac{\left| \varvec{R}\right| }{2} \left[ \tilde{p}_{\varvec{\psi }}(\varvec{v} - \varvec{R}\tilde{\varvec{\eta }_r} \mid \varvec{N} = \varvec{n}) + \tilde{p}_{\varvec{\psi }}(\varvec{v} + \varvec{R}\tilde{\varvec{\eta }_r} \mid \varvec{N} = \varvec{n})\right] }{g(\tilde{\varvec{\eta }}_r)}, \end{aligned}$$

where $\{\varvec{\psi ^*}_1, \ldots , \varvec{\psi ^*}_{D_1}\}$ are $D_1$ draws from $p_{\varvec{\psi }}(\varvec{\psi } \mid \varvec{N} = \varvec{n})$ and $\{\varvec{\tilde{\eta }}_1, \ldots , \varvec{\tilde{\eta }}_{D_2}\}$ are $D_2$ draws from the proposal distribution $g(\varvec{\eta })$. Furthermore, $\tilde{p}_{\varvec{\psi }}(\varvec{\psi }\mid \varvec{N} = \varvec{n})$ denotes the un-normalized posterior density of the unbounded posterior samples; it is therefore written in terms of $\varvec{\xi }_{\text {trans}}$ and is adjusted by the Jacobian term:^11^

20 $$\begin{aligned} \tilde{p}_{\varvec{\psi }}(\varvec{\psi }\mid \varvec{N} = \varvec{n})= & {} \prod _{i = 1}^{I} \left[ \prod ^K_{k=1} \Bigg \{{\frac{J_k!}{n_{ik1}! \times n_{ik2}! \times ... \times n_{ikL_k}!}} \prod ^{L_k}_{l=1}\left[ \text {Pr}(C_{kl} \mid \varvec{\mu }, \varvec{\xi }_{\text {trans}}, \varvec{\omega }_i)\right] ^{n_{ikl}}\Bigg \}\right] \nonumber \\&\quad \times \frac{\Gamma _P(\frac{\nu + I}{2})}{\Gamma _P(\frac{\nu }{2})} \frac{\pi ^{-\frac{I P}{2}}}{\left| { \varvec{\Omega }^\top \varvec{\Omega }+ {\varvec{I}_P}}\right| ^{\frac{\nu + I}{2}}} \times \left( 2\pi \right) ^{-\frac{P}{2}} \exp \bigg \{-\frac{1}{2} \varvec{\mu }^\top \varvec{\mu }\bigg \}\nonumber \\&\quad \times \left( 2\pi \right) ^{-\frac{P}{2}} \exp \bigg \{-\frac{1}{2} \varvec{\xi }_{\text {trans}}^\top \varvec{\xi }_{\text {trans}}\bigg \}. \end{aligned}$$

Note that rewriting the expected value in terms of $\tilde{p}_{\varvec{\psi }}(\varvec{\psi }\mid \varvec{N} = \varvec{n})$ is only a technical nicety. This approach is identical to applying the Warp-III transformation to the posterior samples and then using the iterative scheme with the warped posterior density and a multivariate standard normal proposal distribution.

## Empirical Examples

### Example 1: Nested Model Comparison

We re-analyzed the pair-clustering data set reported in Riefer, Knapp, Batchelder, Bamber, and Manifold (2002) using the hierarchical latent-trait approach.^12^ Experiment 4 examined the memory of patients with brain damage due to prolonged alcoholism in comparison with a control group of alcoholic patients without indications of brain damage. The participants attempted to memorize the same list of 20 categorically related word pairs in a series of six study-test trials.^13^ For demonstration purposes, we focused on the free recall performance of the 21 control participants. Specifically, we investigated whether the model parameters change from the first to the second trial indicating a change in the storage and retrieval processes as a function of practice using posterior model probabilities and Bayesian model averaging.

#### Model Specification

To model differences in parameters, we augmented Eq. (8) with a parameter vector that captures the difference in parameters between the two trials: $\varvec{\delta } = (\delta _c, \delta _r, \delta _u)$. The probit-transformed parameter vectors of participant *i* for the first trial ($\varvec{\theta }_{1,i}^{'}$) and the second trial ($\varvec{\theta }_{2,i}^{'}$) are then obtained as follows:

21 $$\begin{aligned} \begin{aligned} \varvec{\theta }_{1,i}^{'}&= \; \overbrace{\varvec{\mu } - \frac{\varvec{\delta }}{2}}^{\begin{array}{c} \text {group mean} \\ \text {for first trial} \end{array}} \;\,\,+ \;\;\; \varvec{\xi } \odot \varvec{\omega }_i,\\ \varvec{\theta }_{2,i}^{'}&= \underbrace{\varvec{\mu } + \frac{\varvec{\delta }}{2}}_{\begin{array}{c} \text {group mean} \\ \text {for second trial} \end{array}} + \;\;\; \varvec{\xi } \odot \varvec{\omega }_i. \end{aligned} \end{aligned}$$

For an alternative approach to modeling within-subject differences in model parameters, the reader is referred to Rouder, Lu, Morey, Sun, and Speckman (2008).

Table 1 shows the $2^3 = 8$ nested models that implement the eight sets of possible parameter constraints. $\mathcal {M}_1$ allows all three parameters to vary between trials so that $\varvec{\delta } = (\delta _c, \delta _r, \delta _u)$. In contrast, $\mathcal {M}_8$ posits that none of the parameters vary between trials so that $\varvec{\delta } = (0, 0, 0)$. Models $\mathcal {M}_2$ to $\mathcal {M}_7$ are between these extremes and allow either one or two parameters to vary between trials.

**Table 1:** Overview of the eight nested models for the analysis of the first two trials of the pair-clustering data set reported in Riefer et al. (2002).

Free parameters	Model							
	$\mathcal {M}_1$	$\mathcal {M}_2$	$\mathcal {M}_3$	$\mathcal {M}_4$	$\mathcal {M}_5$	$\mathcal {M}_6$	$\mathcal {M}_7$	$\mathcal {M}_8$
*c*	$\checkmark$		$\checkmark$	$\checkmark$		$\checkmark$		
*r*	$\checkmark$	$\checkmark$		$\checkmark$			$\checkmark$	
*u*	$\checkmark$	$\checkmark$	$\checkmark$		$\checkmark$			

Note. $\mathcal {M}_1$ allows all three parameters to vary between trials, and $\mathcal {M}_8$ posits that none of the parameters vary between trials. Models $\mathcal {M}_2$ to $\mathcal {M}_7$ are between these extremes.

We used independent zero-centered normal priors for the components of $\varvec{\delta }$. We explored a narrow ($\sigma ^\text {narrow}_{\delta } \approx 0.52$), medium ($\sigma ^\text {medium}_{\delta } \approx 0.84$), and a wide ($\sigma ^\text {wide}_{\delta } \approx 1.28$) zero-centered normal prior to assess the sensitivity of the results to the width of the test-relevant prior distribution. As shown in Supplemental Materials, the standard deviations $\sigma _{\delta }$ were chosen to correspond to small, medium, and large effects on the probability scale centered around 0.5. Priors for the remaining parameters followed the specification described earlier.

We estimated the posterior distribution of the model parameters using JAGS by adapting the script provided by Matzke et al. (2015). The JAGS code is available in Supplemental Materials. We ran three MCMC chains with overdispersed start values, discarded the first 4000 posterior samples as burn in, and retained only every 20th sample to reduce autocorrelation. Results reported below are based on a total of 90,000 posterior samples. Convergence of the MCMC chains was assessed by visual inspection and the $\hat{R}$ statistic ($\hat{R}<1.05$ for all parameters; Gelman & Rubin, 1992).

**Fig. 3:**
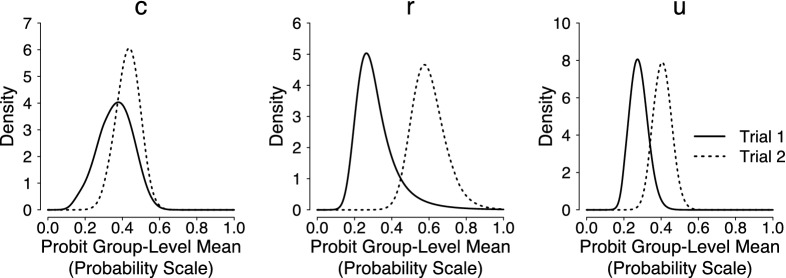
Posterior distributions of the probit group-level means (plotted on the probability scale) from the full model $\mathcal {M}_1$ for the analysis of the first two trials of the pair-clustering data reported in Riefer et al. (2002). The solid lines correspond to the posteriors for the first trial and the dotted lines to the posteriors for the second trial. Available at https://tinyurl.com/y9a33l4t under CC license https://creativecommons.org/licenses/by/2.0/.

Figure 3 shows the resulting posterior distributions of the probit group-level means from the full model $\mathcal {M}_1$; the parameters were transformed back to the probability scale. The posteriors were computed using the medium prior setting ($\sigma ^\text {medium}_{\delta }$)—results obtained with the narrow and wide prior were highly similar and are not displayed. The plot of the posterior distributions based on the alternative prior choice for the elements of $\varvec{\xi }$ (i.e., uniform priors with upper bound $\xi _{\text {max}} = 2$ instead of $\xi _{\text {max}} = 10$) was visually almost indistinguishable from the one presented here and has hence been relegated to Supplemental Materials. The cluster-storage *c* parameter did not change substantially, whereas the storage-retrieval *u*, and especially the cluster-retrieval *r* parameter, seemed to increase from the first trial to the second.

#### Computing Marginal Likelihoods with Warp-III

Equation (20) was adjusted to include the relevant prior distributions for the elements of $\varvec{\delta }$. For each model, we split the 90,000 posterior samples in two equal parts (first and second half of the iterations per chain) and used the first part for estimating $\varvec{R}$ and $\varvec{v}$ and the second part for the iterative updating scheme in Eq. (15) (Overstall & Forster, 2010). Hence, $D_1 = D_2 = 45,000$. To assess the accuracy of the resulting estimates, we repeated this procedure 50 times.^14^ We implemented the procedure in R (R Core Team, 2016). For efficiency, we parallelized the computations, and coded the computationally intensive elements in efficient C++ code which was called from within R using Rcpp (Eddelbuettel et al., 2011). Using a standard personal computer and four CPU cores, computing the marginal likelihood for each repetition took less than one minute per model. The code is available in Supplemental Materials.

**Fig. 4:**
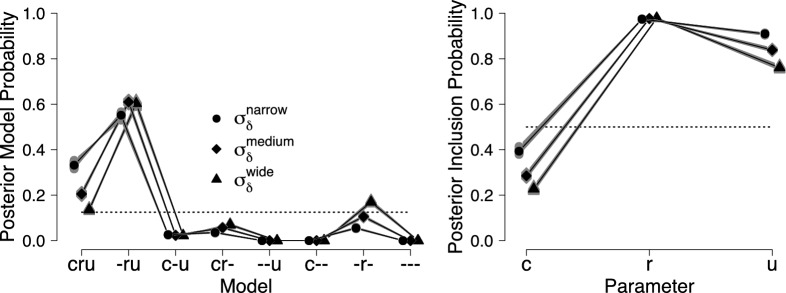
Posterior model probabilities (left panel) and posterior inclusion probabilities (right panel) for the analysis of the first two trials of the pair-clustering data reported in Riefer et al. (2002) obtained with Warp-III bridge sampling. In the left panel, the *x*-axis indicates which parameters were allowed to vary from the first to the second trial (e.g., $c-u$ corresponds to $\mathcal {M}_3$ where *r* was fixed between trials). Gray symbols show the results of the 50 repetitions, and black symbols display the posterior model probabilities and posterior inclusion probabilities that are based on the median of the 50 estimated log marginal likelihoods. Circles show results obtained with the narrow prior, diamonds with the medium prior, and triangles with the wide prior. The dotted lines show the prior model probabilities and prior inclusion probabilities. Available at https://tinyurl.com/yaxbj9o6 under CC license https://creativecommons.org/licenses/by/2.0/.

#### Posterior Model Probabilities

To formally quantify evidence for the differences in parameters, we computed the posterior model probabilities of the eight models using the marginal likelihoods obtained with Warp-III. We assumed that all models were equally likely a priori. The left panel of Fig. 4 shows the posterior model probabilities for the narrow, medium, and wide prior settings. The plot of the posterior model probabilities based on the alternative prior choice for the elements of $\varvec{\xi }$ (i.e., uniform priors with upper bound $\xi _{\text {max}} = 2$ instead of $\xi _{\text {max}} = 10$) was visually almost indistinguishable from the one presented here and has hence been relegated to Supplemental Materials. Formal model comparison confirmed the results of the visual inspection of the posterior distributions shown in Fig. 3: $\mathcal {M}_2$, the model that allows for a difference in *r* and *u*, received the most support from the data. As expected, the width of the test-relevant prior $\varvec{\delta }$ influenced the value of the marginal likelihood, but it did not change the conclusions qualitatively. Warp-III provided accurate estimates of the posterior model probabilities as indicated by the small variability across the 50 repetitions (i.e., gray symbols). For this nested example, the posterior model probabilities can be also obtained using the Savage–Dickey density ratio representation of the Bayes factor (Dickey & Lientz, 1970; Wagenmakers, Lodewyckx, Kuriyal, & Grasman, 2010). As shown in Supplemental Materials, the Savage–Dickey procedure resulted in posterior model probabilities that were highly similar to the ones obtained with Warp-III.

#### Bayesian Model Averaging

Bayesian model averaging does not require researchers to commit to a single “best” model; it allows researchers to acknowledge uncertainty about the choice of the correct model (e.g., Hoeting et al., 1999; Rouder et al., 2017). This is achieved by considering the posterior inclusion probabilities of the parameters. Posterior inclusion probabilities quantify the model-averaged evidence for a change in a given parameter; they can be obtained by summing the posterior model probabilities of the models that allow the parameter to differ between the trials. For instance, the posterior inclusion probability of the *c* parameter is obtained by summing the posterior model probabilities of $\mathcal {M}_1$, $\mathcal {M}_3$, $\mathcal {M}_4$, and $\mathcal {M}_6$. Posterior inclusion probabilities are then compared to the prior inclusion probabilities, in this case 0.5, which are obtained in an analogous manner but based on the prior model probabilities.^15^ The right panel of Fig. 4 shows the posterior inclusion probabilities for the three prior settings. The plot of the posterior inclusion probabilities based on the alternative prior choice for the elements of $\varvec{\xi }$ (i.e., uniform priors with upper bound $\xi _{\text {max}} = 2$ instead of $\xi _{\text {max}} = 10$) was visually almost indistinguishable from the one presented here and has hence been relegated to Supplemental Materials. The posterior inclusion probabilities of the *r* and *u* parameter are higher than the prior inclusion probabilities, indicating evidence for a difference in these parameters between trials. In contrast, the posterior inclusion probability of *c* is lower than the corresponding prior inclusion probability, indicating evidence for invariance between the trials. As before, the width of the $\varvec{\delta }$ prior does not change the conclusions qualitatively.

#### Substantive Contribution

The data from Riefer et al. (2002) have been analyzed in a number of articles. The original article analyzed the aggregated data (an approach known to suffer from limitations in case there is heterogeneity across participants, e.g., Klauer, 2006) and considered the *p* values of $G^2$ statistics to investigate whether parameters differ across trials. Smith and Batchelder (2010) re-analyzed a subset of the data using the hierarchical beta-MPT model (which specifies group-level beta distributions and thus differs from the latent-trait approach that we used).^16^ To investigate whether parameters differ across trials, Smith and Batchelder (a) considered the posterior distribution of the difference between trials for the group-level mean parameters and (b) ran a classical paired sample *t* test on the individual-level parameter estimates. These approaches, however, do not allow one to quantify evidence for an invariance (i.e., a simpler model where some parameters do not differ across trials) on a continuous scale in a systematic way and, crucially, they do not allow one to disentangle “absence of evidence” (i.e., the data are uninformative) and “evidence of absence” (i.e., the data support a simpler model).^17^ These shortcomings can be addressed by computing Bayes factors and posterior model and posterior inclusion probabilities. “Absence of evidence” can be inferred from Bayes factors close to one and posterior model and posterior inclusion probabilities close to the corresponding prior probabilities. In contrast, “evidence of absence” can be inferred from large Bayes factors in favor of the simpler model, and in situations when the posterior model probability of the simpler model is the highest or when the posterior inclusion probability is smaller than the prior inclusion probability.

Our Bayesian re-analysis suggests that there is strong evidence that the probability of retrieving word pairs that have been stored as a cluster (i.e., *r*) changed from the first to the second trial. Furthermore, there is evidence that the probability of storing and retrieving words that have not been stored as a cluster (i.e., *u*) differed between the two trials. Crucially, our approach also allowed us to conclude that there is some evidence that the probability of storing a word pair as a cluster (i.e., *c*) did *not* change from the first to the second trial (although this evidence is not that pronounced since the posterior inclusion probability for a difference in *c* is—depending on the prior choice—relatively close to the prior inclusion probability of .5). Another key improvement in our analysis over the above-mentioned analyses is the use of Bayesian model averaging. In this example, $\mathcal {M}_2$ received the highest posterior probability; however, $\mathcal {M}_1$ also received substantive posterior probability. Therefore, selecting a single best model (i.e., $\mathcal {M}_2$) and basing final inference solely on this model might be suboptimal at best and misleading at worst. In contrast, when using the model-averaged posterior inclusion probabilities for drawing conclusions about which parameters differ between trials, one takes into account all models under consideration according to their plausibilities in light of the observed data.

Finally, note that one might argue that this data set is relatively small and is thus uninformative. However, one strength of the Bayesian approach is that it allows one to quantify whether the data are informative or not. For this example, the Bayesian results suggest that the data are in fact informative which is indicated by posterior model/inclusion probabilities that are quite different from the corresponding prior probabilities.

### Example 2: Non-Nested Model Comparison

We re-analyzed data from Experiment 2 reported by Fazio et al. (2015) who investigated the influence of knowledge on the illusory truth effect. The illusory truth effect refers to the phenomenon that, in the absence of knowledge about the truth status of a statement, repeated statements are easier to process and are judged more truthful than new statements. Fazio et al., however, provided evidence that participants tend to rely on the ease of processing (i.e., fluency) even when they have knowledge about the statement.

We re-analyzed data from 39 participants who indicated the truthfulness (i.e., “true”/“false”) of 176 statements, half of which were true and half of which were false. Half of the statements were likely to be known according to general knowledge norms (“known” statements) and half of them were likely to be unknown (“unknown” statements). An example of a true known statement is “The Pacific Ocean is the largest ocean on Earth.” An example of a false unknown statement is “Billy the Kid’s last name is Garrett.” To manipulate fluency, half of the statements were presented twice, once in the exposure phase and once in the truth-rating phase, whereas the other half was only presented in the truth-rating phase. Hence, the experiment had a 2 (truth status: true vs. false) $\times$ 2 (assumed knowledge: known vs. unknown) $\times$ 2 (repetition: repeated vs. not repeated) balanced within-subject design, and each cell of the design featured 22 statements.

**Fig. 5:**
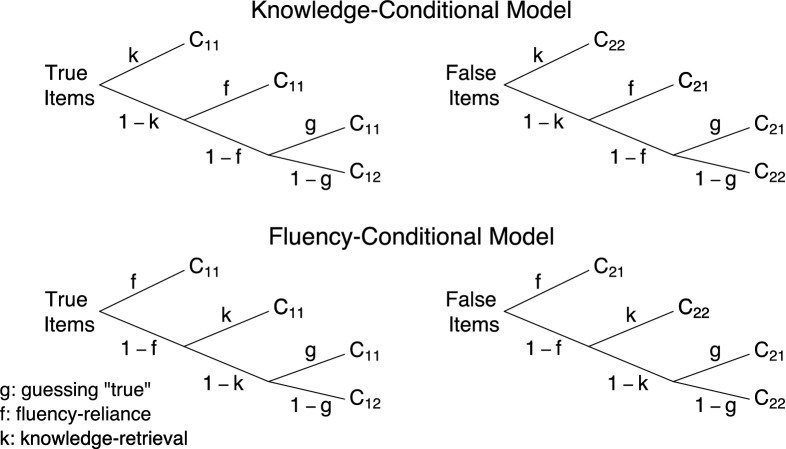
Knowledge-conditional (top panel) and fluency-conditional (bottom panel) MPTs. Available at https://tinyurl.com/ya8sovfr under CC license https://creativecommons.org/licenses/by/2.0/.

#### Model Specification

Fazio et al. (2015) constructed two MPTs to study the illusory truth effect. The knowledge-conditional model depicted in the top panel of Fig. 5 assumes that participants rely on knowledge when assessing truthfulness and only rely on fluency when they are unable to retrieve knowledge about the statement. Parameter *k* represents the probability of retrieving knowledge about the statement from memory. If knowledge is retrieved, participants are assumed to give the correct response (i.e., “true” for true statements and “false” for false statements). If no knowledge is retrieved with probability $1 - k$, participants rely on fluency with probability *f* and respond “true.” If participants do not rely on fluency with probability $1 - f$, they guess “true” with probability *g* and “false” with probability $1 - g$. Responses to true statements are scored into the categories $C_{11}$ (correct “true” response) and $C_{12}$ (incorrect “false” response). Responses to false statements are scored into the categories $C_{21}$ (incorrect “true” response) and $C_{22}$ (correct “false” response). In contrast, the fluency-conditional model depicted in the bottom panel reflects the notion that participants mainly rely on fluency and only use knowledge in the absence of fluency. The models feature the same set of parameters, but they assume a different conditional probability structure.

For each model, we replicated the two subtrees four times (i.e., a total of eight subtrees per model) to accommodate the design of the experiment: The first replicate corresponded to known true and false statements that were not repeated, the second to known true and false statements that were repeated, the third to unknown true and false statements that were not repeated, and the fourth to unknown true and false statements that were repeated. Following Fazio et al. (2015), we used separate knowledge parameters for known ($k_k$) and unknown ($k_u$) statements, and separate fluency parameters for repeated statements ($f_r$) and statements shown only once ($f_n$). The guessing parameter *g* was constrained to be equal across the four replicates. We implemented the models within the hierarchical latent-trait approach, using the prior specifications described earlier.

We estimated the posterior distribution of the model parameters using JAGS, ran three MCMC chains with overdispersed start values, discarded the first 4000 posterior samples as burn in, and retained only every 50th sample. Results reported below are based on a total of 180, 000 posterior samples. The posterior distributions of the group-level mean parameters are displayed in Supplemental Materials.

#### Computing Bayes Factors with Warp-III

For each model, we split the 180, 000 posterior samples in two equal parts (first and second half of the iterations per chain) and used the first part for estimating $\varvec{R}$ and $\varvec{v}$ and the second part for the iterative updating scheme in Eq. (15) ($D_1 = D_2 = 90,000$). Using a standard personal computer and four CPU cores, computing the marginal likelihood took approximately three minutes per model.

The resulting marginal likelihoods were used to compute the Bayes factor in favor of the fluency-conditional model over the knowledge-conditional model. To assess the accuracy of the resulting Bayes factor, we repeated this procedure 50 times. Estimates of the Bayes factor ranged from $1.3 \times 10^{42}$ to $3.6 \times 10^{43}$ in favor of the fluency-conditional model. Estimates of the Bayes factor based on the alternative prior choice for the elements of $\varvec{\xi }$ (i.e., uniform priors with upper bound $\xi _{\text {max}} = 2$ instead of $\xi _{\text {max}} = 10$) ranged from $1.7 \times 10^{41}$ to $1.7 \times 10^{43}$ in favor of the fluency-conditional model. In line with the conclusion drawn by Fazio et al. (2015) based on the $G^2$ statistic, this result provides overwhelming evidence in favor of the fluency-conditional model.^18^

**Fig. 6:**
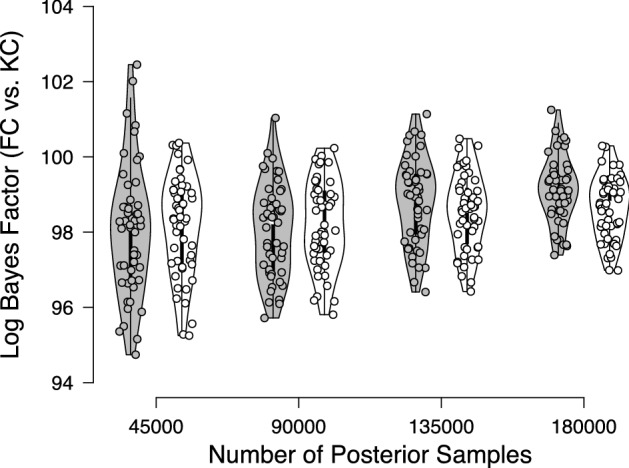
Log Bayes factor estimates in favor of the fluency-conditional (FC) model over the knowledge-conditional (KC) model as a function of the number of posterior samples. The Warp-III estimates are displayed in white, and the estimates based on the simpler multivariate normal approach are displayed in gray. Available at https://tinyurl.com/ydbfev7w under CC license https://creativecommons.org/licenses/by/2.0/.

Figure 6 displays the Warp-III Bayes factor estimates (on the log scale) in white as a function of the number of posterior samples used in the bridge sampling procedure.^19^ As a comparison, the estimates based on the simpler multivariate normal bridge sampling approach are displayed in gray. As the number of posterior samples increases, the Bayes factor estimates become more precise. For this particular example, it is apparent that the Warp-III estimates are less variable than the estimates based on the simpler multivariate normal approach.

#### Substantive Contribution

The authors of the original article analyzed the aggregated data (again, an approach known to be suboptimal in case there is heterogeneity across participants) and considered the $G^2$ statistics with corresponding *p* values. Based on the fact that the knowledge-conditional model had a larger, significant $G^2$ statistic compared to the fluency-conditional model that had a lower, nonsignificant $G^2$ statistic, the authors concluded that the knowledge-conditional model fit the data poorly and the fluency-conditional model fit the data well. Therefore, the authors favored the fluency-conditional model based on two binary accept–reject decisions. This makes it difficult to gauge the degree of support that the data provide in favor of the fluency-conditional model. The Bayes factor may be 10, or 100, or 1000—these are very different levels of evidence. In fact, our analysis shows that the Bayes factor is about $1.3 \times 10^{42}$–$3.6 \times 10^{43}$ in favor of the fluency-conditional model, which represents an overwhelming amount of evidence.

It could be argued that, since the compared models have the same number of parameters, comparing $G^2$ statistics may result in choosing the same model as based on considering AIC or BIC. AIC is asymptotically equivalent to cross-validation (Stone, 1977) which is known to be inconsistent in the sense that, when the number of observations goes to infinity, the data-generating model will not be chosen with certainty (Shao, 1993). In contrast, when using Bayes factors, model selection consistency is generally fulfilled (Bayarri, Berger, Forte, & García-Donato, 2012). Although the BIC is a rough approximation of the Bayes factor, we believe that it is better to compute proper Bayes factors which are transparent with respect to the prior assumptions.

Finally, one might argue again that this data set is relatively small and is thus uninformative. However, the resulting Bayes factor is very different from 1, indicating that the data are in fact highly informative with respect to adjudicating between the fluency-conditional and the knowledge-conditional models.

## Discussion

Bayesian hierarchical techniques for MPT modeling are increasingly popular. Current hierarchical MPT approaches, however, do not incorporate Bayesian model comparison methods based on Bayes factors and posterior model probabilities, possibly because of the computational challenges associated with the evaluation of the marginal likelihood. In this article, we addressed this challenge and showed how Warp-III bridge sampling can be used to obtain accurate and stable estimates of the marginal likelihood of hierarchical MPTs. We applied the method to model comparison problems from two published studies and illustrated how the marginal likelihood can be used for Bayesian model averaging and for the computation of the Bayes factor.

Our examples highlighted that Bayesian model comparison based on posterior model/inclusion probabilities and Bayes factors allows researchers to disentangle between “absence of evidence” and “evidence of absence.” Note that it is crucial in all stages of cognitive model development, validation, and application that one is able to quantify evidence in favor of invariances (i.e., “evidence of absence”) in a coherent and systematic way. For model development and validation, it is important to show that certain experimental manipulations selectively influence only a subset of the model parameters, whereas the remaining parameters are unaffected (i.e., selective influence studies). Once a cognitive model has been established as a valid measurement tool, it can be used, for instance, to investigate which subprocesses are targeted by new experimental manipulations or which subprocesses differ or do not differ in clinical subpopulations (cognitive psychometrics; e.g., Riefer et al., 2002). In these applications, it is important to be able to quantify evidence for a difference but, crucially, also for an invariance since one might wish to make statements of the form “there is evidence that retrieval processes are not affected.”

There are often a number of different candidate models for the analysis of observed data. In Example 1, we demonstrated how Bayesian model averaging can be used to draw conclusions that fully take into model uncertainty. In our opinion, Bayesian model averaging is an extremely powerful approach and, to the best of our knowledge, it is currently not used in the context of hierarchical MPTs and cognitive modeling more generally. We believe that attending researchers to this approach and providing the computational tools to facilitate its application (i.e., Warp-III) is one of the key contributions of this work.

Our examples illustrated that Warp-III is relatively straightforward to implement once posterior samples from the models have been obtained with MCMC sampling. Another advantage of Warp-III bridge sampling is its relative speed. In our experience, the Warp-III procedure requires much less computational time than the MCMC sampling from the posterior. One of the crucial determinants of the computational time of Warp-III is how long it takes to evaluate the un-normalized posterior density. To maximize speed for our applications, we implemented the un-normalized posterior density functions in C++ code called from within R via Rcpp (Eddelbuettel et al., 2011). Compared to a simpler bridge sampling version which only matches the first two moments of the proposal and the posterior (e.g., Overstall & Forster, 2010), Warp-III is expected to take about twice as long for a fixed number of samples due to the mixture representation of the warping procedure which requires evaluating the un-normalized posterior twice as often as for the simpler bridge sampling version. However, Warp-III is also expected to be more accurate in case the posterior is skewed which means there might be a speed–accuracy trade-off.

Despite its computational simplicity, Warp-III should not be applied blindly. Specifically, as we demonstrated for our empirical examples, it is important to assess the variability of the resulting model comparison measure—such as posterior model probabilities or Bayes factors—by repeating the Warp-III procedure multiple times. When the measure of interest clearly favors a given model, as in our second example, some fluctuation is not necessarily concerning. However, in situations where the fluctuation influences which model is favored, researchers should either increase the number of posterior and proposal samples to decrease the variability of the estimate, or, if this solution is practically infeasible, they should acknowledge that the estimate does not support firm conclusions about the relative predictive adequacy of the models.

The accuracy of the estimate is governed not only by the number of samples but also by the overlap between the proposal and the posterior distribution. Warp-III attempts to maximize this overlap by matching the mean vector, covariance matrix, and the skew of the two distributions. However, in case the posterior distribution exhibits multiple modes, the overlap may not be sufficiently close. Researchers should carefully check whether multimodalities occur in their application. If this is the case, repeated runs of the Warp-III procedure could be used to obtain an impression of the stability of the estimate. Nevertheless, it should be kept in mind that Warp-III is not designed for multimodal posterior distributions and results should be interpreted with caution. The development of bridge sampling procedures for multimodal posterior distributions is currently ongoing (e.g., Frühwirth–Schnatter, 2004; Wang & Meng, 2016). Note, however, that this is not a very severe limitation of the Warp-III method, since posterior distributions are unimodal in many models used in psychology—they even converge to normal distributions under specific conditions (Dawid, 1970).

Relatedly, note that we use the unscaled effects $\varvec{\omega }_i$ and the scaling parameters $\varvec{\xi }$ directly in the bridge sampling procedure—but technically, these are only identified jointly. Therefore, MCMC chains for these parameters may look irregular and exhibit, for instance, multiple modes, decreasing the efficiency of the Warp-III procedure as mentioned above. Although this was not the case for our applications, we advise researchers to carefully monitor the MCMC chains of the unidentified unscaled effects and scaling parameters.

On a more theoretical note, as Eq. (3) illustrates, Bayesian model comparison is sensitive to the choice of the prior distribution. We relied on relatively standard priors for the group-level parameters, but also established the robustness of our conclusions with a series of sensitivity analyses (see also Supplemental Materials). Nevertheless, we do not suggest that our prior choices should be considered as the gold standard for model comparison in hierarchical MPTs. Several approaches are available for specifying theoretically justified prior distributions for cognitive models (Lee & Vanpaemel, 2018; see also Heck & Wagenmakers, 2016, for specifying order constraints in MPTs). We believe that the increasing popularity of hierarchical MPTs will enable researchers to specify informative paradigm-specific and model-specific prior distributions based on experience with the models (e.g., typical parameter ranges and effect sizes). The dependency on the prior is sometimes considered as a weakness of Bayes factor model comparisons (e.g., Aitkin, 2001). Some researchers and statisticians even conclude that due to this reason, the use of Bayes factors is not recommended (e.g., Gelman et al., 2014, chapter 7.4).^20^ In contrast, we believe that the ability to incorporate prior knowledge is an advantage of Bayesian inference; we consider the prior as integral part of the model which should be chosen just as carefully as the likelihood (e.g., Vanpaemel, 2010). Ideally, researchers should preregister their priors before data collection (Chambers, 2013, 2015) to ensure that these are used to express genuine prior knowledge and not to increase researchers’ degrees of freedom in obtaining the desired results. Note that we are not the first to advocate a Bayesian approach to hierarchical MPTs. However, to the best of our knowledge, we are the first who advocate Bayesian model comparison using posterior model/inclusion probabilities and Bayes factors and provide the tools to compute these quantities for hierarchical MPTs. Equipped with a feasible approach for computing the relevant quantities for Bayesian model comparison, one could, in principle, specify an informed prior for the models themselves in addition to the specification of the parameter prior. This way one could incorporate prior knowledge about how likely each model is or one could, if desired, incorporate a penalty for multiple comparisons as described in Scott and Berger (2010).

Although we focused exclusively on latent-trait MPTs, Warp-III is not limited to the latent-trait approach or other hierarchical MPTs, such as the beta-MPT (Smith & Batchelder, 2010) or the crossed random effects approach (Matzke et al., 2015). Warp-III may be used to compute the marginal likelihood for a large variety of cognitive models. For instance, the simple multivariate normal bridge sampling approach has been recently applied to hierarchical reinforcement learning models (Gronau et al., 2017). We believe that Warp-III may be especially useful for so-called sloppy models with highly correlated parameters (Brown & Sethna, 2003), including but not limited to race models of response times, which often yield skewed posterior distributions (e.g., Brown & Heathcote, 2008; Matzke, Love, & Heathcote, 2017). The Warp-III methodology also lends itself to model comparison in extensions of hierarchical cognitive models that impose on the model parameters a statistical structure such as a linear regression, factor analysis, or analysis of variance (e.g., Boehm, Steingroever, & Wagenmakers, 2017; Heck et al., 2018a; Turner, Wang, & Merkle, 2017; Vandekerckhove, 2014). The application of Warp-III to complex experimental designs is ongoing work in our laboratory.

Although Warp-III is a general procedure for computing the marginal likelihood, depending on the situation, other approaches may be better suited for the model comparison problem at hand. If researchers focus on non-hierarchical implementations of cognitive models, importance sampling may be an easier solution, particularly in the context of MPTs (Vandekerckhove et al., 2015). If the focus is on nested models, the Savage–Dickey density ratio is an easier and faster alternative. Lastly, if the number of models under consideration is very large, Reversible Jump MCMC (Green, 1995) might be the appropriate choice. Nevertheless, we believe that in most applications of hierarchical cognitive models, the research question concerns the comparison of a limited set of possibly non-nested models. In these situations, Warp-III provides a straightforward and accurate method for computing the marginal likelihood for a wide range of complex models.

## Data Availability Statement

The datasets analyzed during the current study are available on the Open Science Framework: https://osf.io/rycg6/.

## Electronic supplementary material

Below is the link to the electronic supplementary material.

Supplementary material 1 (pdf 897 KB)

## Footnotes

1 The interested reader is referred to Plieninger and Heck (2018) for a comparison of these model classes.

2 Note that posterior model probabilities can also be obtained using information criteria (e.g., Burnham & Anderson, 2002; Wagenmakers & Farrell, 2004).

3 For details on the predictive interpretation of the marginal likelihood, see Supplemental Materials available at https://osf.io/rycg6/.

4 Hu (2001), Heck and Erdfelder (2016), and Heck, Erdfelder, and Kieslich (2018b) proposed extensions that also incorporate response times.

5 Bayesian hierarchical models can be also used to account for heterogeneity in items instead of participants.

6 We omit conditioning on the model for enhanced legibility.

7 Bridge sampling in its original form has been proposed to estimate a *ratio* of normalizing constants. This approach, however, becomes challenging and inefficient in case the two models have different parameter spaces (e.g., non-nested comparisons), and potentially very little overlap between the posterior distributions. For these cases, it may be easier and more efficient to compute each normalizing constant separately (e.g., DiCiccio et al., 1997; Overstall & Forster, 2010). This ensures that the two relevant distributions (i.e., proposal and posterior) for each of the separate bridge sampling applications are close to each other yielding an efficient estimator. Therefore, we recommend computing each normalizing constant separately to enable application of the method to a wide range of model comparison scenarios.

8 Specifically, we used the median effective sample size across all posterior components.

9 In our experience, the exact value of the initial guess typically does not have a lasting influence on the resulting estimate. Nevertheless, good initial values may lead to faster convergence. For implementation details, see Gronau et al. (2017), especially Appendix B.

10 As before, the probit transformation is defined component-wise.

11 Note that $\xi _{\text {max}}$ drops out of the expression because it cancels with the first term of the Jacobian. Implicitly, however, it still influences the marginal likelihood because it appears in the transformation equation $\varvec{\xi }_{\text {trans}} = \Phi ^{-1}\left( \frac{\varvec{\xi }}{\xi _{\text {max}}}\right)$. It is also needed for evaluating $\text {Pr}(C_{kl} \mid \varvec{\mu }, \varvec{\xi }_{\text {trans}}, \varvec{\omega }_i)$ since in order to obtain the MPT parameters on the probit scale (i.e., Eq. (8)) we need to transform $\varvec{\xi }_{\text {trans}}$ back to $\varvec{\xi }$ via the inverse transformation $\varvec{\xi } = \xi _{\text {max}} \, \Phi \left( \varvec{\xi }_{\text {trans}}\right)$.

12 Data were obtained from https://bayesmodels.com/; see also Lee and Wagenmakers (2013).

13 Riefer et al. (2002) did not administer singletons.

14 We assessed the accuracy of the estimates conditional on the posterior samples, that is, for each repetition, we used the same posterior samples but generated new samples from the proposal distribution. Whenever feasible, it may be advantageous to also generate new posterior samples in each repetition.

15 The change from prior inclusion *odds* to posterior inclusion *odds* can also be quantified by means of an inclusion Bayes factor (not reported).

16 Note that this data set has been also analyzed in Lee and Wagenmakers (2013, chapter 14). In this case, the hierarchical latent-trait approach was used; however, no explicit model comparison or hypothesis testing was conducted.

17 Note also that it is well known that the two-step procedure (b) used by Smith and Batchelder can yield biased conclusions (Boehm, Marsman, Matzke, & Wagenmakers, 2018).

18 Although the Bayes factor indicates overwhelming evidence in favor of the fluency-conditional model, it should be kept in mind that the Bayes factor quantifies the evidence of two models relative to each other. In practice, researchers should also check that the model that is favored by the Bayes factor provides an adequate fit to the observed data (e.g., Steingroever, Wetzels, & Wagenmakers, 2014).

19 Posterior sample sizes smaller than 180,000 were obtained by considering only a subset of the 180,000 posterior samples for each model (i.e., no new posterior samples were obtained). Note that the same posterior sample sizes were used for the Warp-III and the simpler multivariate normal approach, but the results of the two methods are displayed with an offset to avoid overlapping symbols. Plots for each model’s marginal likelihood estimates are presented in Supplemental Materials.

20 Another objection is that Bayes factors are often used to compare nested models where certain values of continuous parameters are treated as “special” (since the parameters are fixed to these values). These researchers often favor continuous model expansion instead (e.g., Gelman et al., 2014, chapter 7.4; Gelman & Rubin, 1995).
