# Supplementary material for: A Simple Method for Comparing Complex Models: Bayesian Model Comparison for Hierarchical Multinomial Processing Tree Models Using Warp-III Bridge Sampling
Source: Psychometrika. 2018 Nov 27;84(1):261–84. doi: 10.1007/s11336-018-9648-3 (PMC6684497; doi:10.1007/s11336-018-9648-3)
Supplement: Supplementary file 1 — Supplementary material 1 (pdf 897 KB) [file 11336_2018_9648_MOESM1_ESM.pdf]

A Simple Method for Comparing Complex Models: Bayesian Model Comparison for Hierarchical Multinomial Processing Tree Models Using Warp-III Bridge Sampling (Online Appendix)

Appendix A: Predictive Interpretation of the Marginal Likelihood

Here we explain the predictive interpretation of the marginal likelihood. Recall that the marginal likelihood is obtained by integrating out the model parameters with respect to the parameters' prior distribution:

$$p(\text{data} \mid \mathcal{M}_i) = \int_{\boldsymbol{\Theta}} p(\text{data} \mid \boldsymbol{\theta}, \mathcal{M}_i)\, p(\boldsymbol{\theta} \mid \mathcal{M}_i) \mathrm{d}\boldsymbol{\theta}. \tag{1}$$

The predictive interpretation of the marginal likelihood is obtained by considering Equation 1 as a function of the data. Hence, we obtain a distribution over data patterns predicted by the model. This distribution is called *prior predictive distribution*. For illustration purposes, Figure 1 displays two exemplary prior predictive distributions. The gray distribution corresponds to a simple model, $\mathcal{M}_1$, which makes relatively precise predictions. In contrast, the shaded distribution corresponds to the predictive distribution of $\mathcal{M}_2$, a more complex model whose predictions are more spread out. Note that these by the models predicted distributions are proper probability distributions and consequently sum to one across all possible data patterns (in case of continuous data, they would integrate to one). Hence, if a model assigns more probability to a certain data pattern (e.g., $\mathcal{M}_1$ assigns more probability to data patterns in the middle of Figure 1), it necessarily needs to assign less probability to other data patterns (e.g., $\mathcal{M}_1$ assigns less probability to data patterns in the left and right parts of Figure 1).

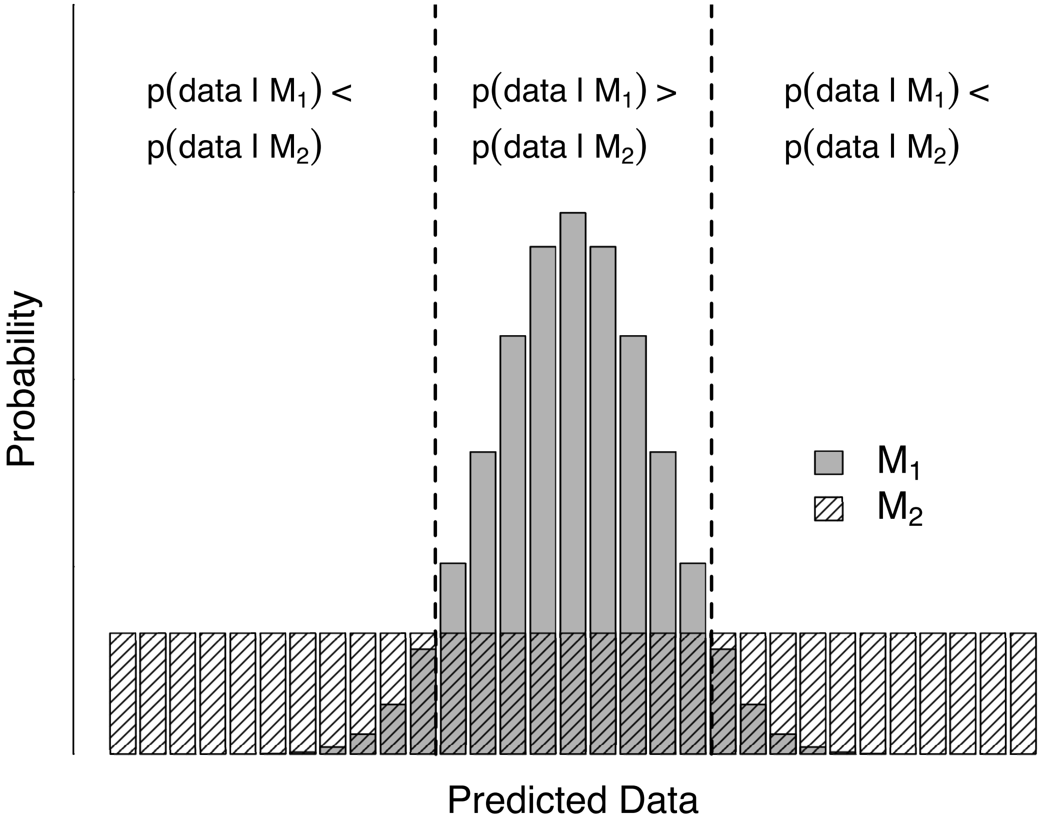

Figure 1: Exemplary prior predictive distributions (i.e., by the models predicted distributions of data patterns) for a simple model ($\mathcal{M}_1$, gray distribution) and a more complex model ($\mathcal{M}_2$, shaded distribution). The dashed vertical lines separate areas of the data space in which one model assigns more mass to data in that subspace than the other model. Figure available at `https://tinyurl.com/yaptsw8b` under CC license `https://creativecommons.org/licenses/by/2.0/`.

The marginal likelihood of a model corresponds to the height of the by the model predicted distribution at the observed data. Consequently, the marginal likelihood of the simpler model $\mathcal{M}_1$ will be larger than the one of the more complex model $\mathcal{M}_2$ if the data fall in the middle area of Figure 1. Thus, when comparing these two models by means of a Bayes factor which corresponds to the ratio of the heights of the predictive distributions at the observed data, one will obtain evidence in favor of the simpler model $\mathcal{M}_1$. This highlights that simpler models that make more precise predictions are rewarded compared to more complex models in case the observed data fall within the predicted area of the data space. However, in case the observed data do not fall within that area (e.g., the outer parts of Figure 1) the more complex model will be supported since its additional complexity is in this case needed.

Appendix B: Alternative Prior for $\boldsymbol{\xi}$

Here we explain how we chose the alternative prior distribution for the components of $\boldsymbol{\xi}$ (i.e., $\xi_p \sim \text{Uniform}(0, \xi_{\max})\, \forall p \in \{1, 2, \ldots, P\}$ where $\xi_{\max} = 2$) based on considering the pattern of implied group-level distributions on the probability scale. The marginal group-level distribution of a single probit MPT parameter θ'_{ip} is the following univariate normal distribution: $\theta'_{ip} \sim \mathcal{N}(\mu_p, \xi_p^2\, q_{pp})$ where q_{pp} denotes the entry in the pth row and pth column of $\boldsymbol{Q}$. A straightforward way of inspecting the implied group-level distributions on the probability scale is to repeatedly draw from the priors for $\boldsymbol{\xi}$, $\boldsymbol{\mu}$, and $\boldsymbol{Q}$ and then transform the resulting normal distributions to the probability scale. Each draw from the priors results

in a separate group-level distribution on the probability scale. To visualize the pattern of implied group-level distributions on the probability scale, each of the resulting group-level distributions is summarized by its mean and standard deviation. These are obtained using the *law of the unconscious statistician.* The group mean $\overline{\theta_{ip}}$ on the probability scale is then obtained by numerically evaluating $\int_{-\infty}^{\infty} \Phi(\theta'_{ip})\ \mathcal{N}(\theta'_{ip}; \mu_p, \xi_p^2\, q_{pp})\mathrm{d}\theta'_{ip}$ where $\mathcal{N}(x; y, z)$ corresponds to the probability density function (pdf) of a normal distribution for x with mean y and variance z (see also Heck, Arnold, & Arnold, 2018). The standard deviation is obtained via $\sqrt{\int_{-\infty}^{\infty} (\Phi(\theta'_{ip}) - \overline{\theta_{ip}})^2\ \mathcal{N}(\theta'_{ip}; \mu_p, \xi_p^2\, q_{pp})\mathrm{d}\theta'_{ip}}$.

Figure 2 displays the pattern of implied group-level distributions on the probability scale for four different prior choices for $\boldsymbol{\xi}$. Each panel is based on drawing 2,000 times from the priors and then plotting the implied group mean and standard deviation on the probability scale as a dot in the scatter plot. The upper-left panel shows the results for the original prior choices by Klauer (2010) who used independent normal priors with mean one and variance 100 for the elements of $\boldsymbol{\xi}$. Furthermore, he used zero-centered normal priors with variance 100 for the elements of $\boldsymbol{\mu}$. The upper-right panel depicts the results for the choices by Matzke, Dolan, Batchelder, and Wagenmakers (2015) who used independent uniform priors with lower bound zero and upper bound 100 for the elements of $\boldsymbol{\xi}$ and standard normal priors for the components of $\boldsymbol{\mu}$. The lower-left panel displays the results for the same prior choice but this time using uniform distributions with upper bound ten; this is the default choice in `TreeBUGS` (Heck et al., 2018). The lower-right panel shows the results for uniform priors with upper bound two. To facilitate interpretation, the solid lines depict for each possible mean the maximal possible standard deviation given by $\sqrt{\overline{\theta_{ip}}(1 - \overline{\theta_{ip}})}$. Figure 2

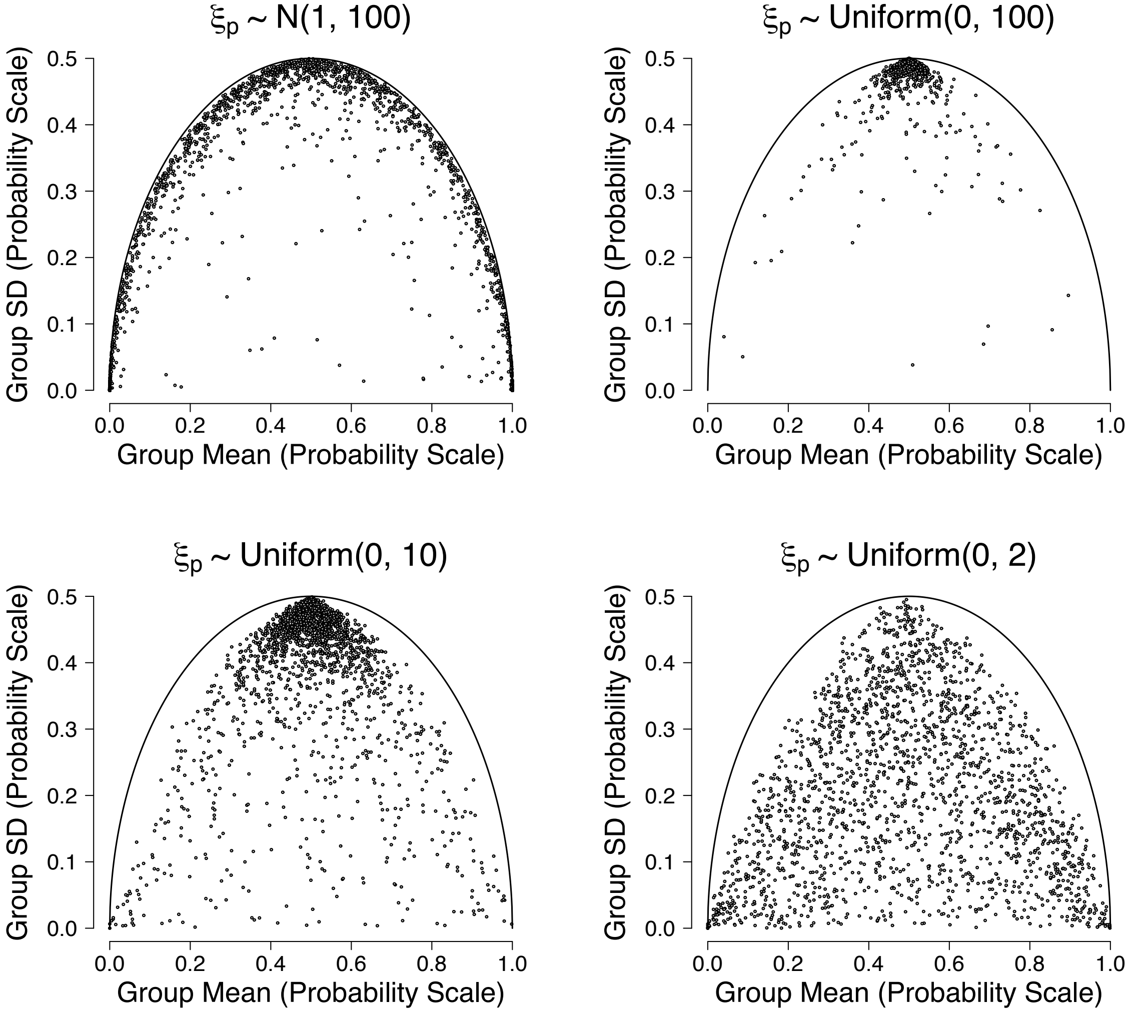

Figure 2: Pattern of implied group-level distributions on the probability scale for four different prior choices for ξ_p. Figure available at `https://tinyurl.com/ybk5hpbm` under CC license `https://creativecommons.org/licenses/by/2.0/`.

highlights that all prior choices except the uniform prior with upper bound two mostly lead to implied group-level distributions with large standard deviations. Furthermore, for the uniform priors with upper bound 100 and ten, the group means are concentrated around 0.5. In contrast, the implied group-level distributions for $\xi_p \sim \text{Uniform}(0, 2)$ appear to cover the space of possible group-level means and standard deviations more evenly. We believe that this is a desirable property which is the reason why we used $\xi_p \sim \text{Uniform}(0, 2)$ as an alternative prior for conducting a prior sensitivity check (reported below).

Appendix C: Integrating Out the Unscaled Covariance Matrix $\boldsymbol{Q}$ Analytically

Here we show in detail how the unscaled covariance matrix $\boldsymbol{Q}$ can be integrated out analytically from the expression for the marginal likelihood. The part of the integral that involves $\boldsymbol{Q}$ is given by:

$$
\begin{aligned}
&\int \prod_{i=1}^{I} p(\boldsymbol{\omega}_i \mid \boldsymbol{Q})\, p(\boldsymbol{Q}) \mathrm{d}\boldsymbol{Q} \\
&= \int \prod_{i=1}^{I} \left[(2\pi)^{-\frac{P}{2}} \left|\boldsymbol{Q}\right|^{-\frac{1}{2}} \exp\left\{ -\frac{1}{2} \boldsymbol{\omega}_i^\top \boldsymbol{Q}^{-1} \boldsymbol{\omega}_i \right\} \right] \frac{1}{2^{\frac{\nu P}{2}} \Gamma_P\left(\frac{\nu}{2}\right)} \left|\boldsymbol{Q}\right|^{-\frac{\nu+P+1}{2}} \exp\left\{ -\frac{1}{2} \operatorname{tr}\left(\boldsymbol{Q}^{-1}\right) \right\} \mathrm{d}\boldsymbol{Q} \\
&= (2\pi)^{-\frac{IP}{2}} \frac{1}{2^{\frac{\nu P}{2}} \Gamma_P\left(\frac{\nu}{2}\right)} \int \left|\boldsymbol{Q}\right|^{-\frac{\nu+I+P+1}{2}} \exp\left\{ -\frac{1}{2} \left[\sum_{i=1}^{I} \boldsymbol{\omega}_i^\top \boldsymbol{Q}^{-1} \boldsymbol{\omega}_i + \operatorname{tr}\left(\boldsymbol{Q}^{-1}\right) \right] \right\} \mathrm{d}\boldsymbol{Q}.
\end{aligned}
$$

Since $\boldsymbol{\omega}_i^\top \boldsymbol{Q}^{-1} \boldsymbol{\omega}_i$ is a scalar and the trace of a scalar is simply the scalar itself, we can rewrite as:

$$(2\pi)^{-\frac{IP}{2}} \frac{1}{2^{\frac{\nu P}{2}} \Gamma_P(\frac{\nu}{2})} \int |\boldsymbol{Q}|^{-\frac{\nu+I+P+1}{2}} \exp\left\{-\frac{1}{2}\left[\sum_{i=1}^{I} \operatorname{tr}\left(\boldsymbol{\omega}_i^\top \boldsymbol{Q}^{-1} \boldsymbol{\omega}_i\right) + \operatorname{tr}\left(\boldsymbol{Q}^{-1}\right)\right]\right\} \mathrm{d}\boldsymbol{Q}.$$

Next, we use the fact that the trace is invariant under cyclic permutations, that is, $\operatorname{tr}(\boldsymbol{ABC}) = \operatorname{tr}(\boldsymbol{BCA}) = \operatorname{tr}(\boldsymbol{CAB})$:

$$(2\pi)^{-\frac{IP}{2}} \frac{1}{2^{\frac{\nu P}{2}} \Gamma_P(\frac{\nu}{2})} \int |\boldsymbol{Q}|^{-\frac{\nu+I+P+1}{2}} \exp\left\{-\frac{1}{2}\left[\sum_{i=1}^{I} \operatorname{tr}\left(\boldsymbol{\omega}_i \boldsymbol{\omega}_i^\top \boldsymbol{Q}^{-1}\right) + \operatorname{tr}\left(\boldsymbol{Q}^{-1}\right)\right]\right\} \mathrm{d}\boldsymbol{Q}.$$

Since $\operatorname{tr}(\boldsymbol{A}+\boldsymbol{B}+\boldsymbol{C}) = \operatorname{tr}(\boldsymbol{A}) + \operatorname{tr}(\boldsymbol{B}) + \operatorname{tr}(\boldsymbol{C})$, we can rearrange as follows:

$$(2\pi)^{-\frac{IP}{2}} \frac{1}{2^{\frac{\nu P}{2}} \Gamma_P(\frac{\nu}{2})} \int |\boldsymbol{Q}|^{-\frac{\nu+I+P+1}{2}} \exp\left\{-\frac{1}{2}\left[\operatorname{tr}\left(\left[\sum_{i=1}^{I} \boldsymbol{\omega}_i \boldsymbol{\omega}_i^\top + \boldsymbol{I}_P\right] \boldsymbol{Q}^{-1}\right)\right]\right\} \mathrm{d}\boldsymbol{Q}.$$

The integrand is in the form of an un-normalized inverse-Wishart distribution, hence, the integral is equal to the normalizing constant of that distribution. We obtain:

$$\begin{aligned} &(2\pi)^{-\frac{IP}{2}} \frac{1}{2^{\frac{\nu P}{2}} \Gamma_P(\frac{\nu}{2})} \frac{2^{\frac{(\nu+I)P}{2}} \Gamma_P(\frac{\nu+I}{2})}{\left|\sum_{i=1}^{I} \boldsymbol{\omega}_i \boldsymbol{\omega}_i^\top + \boldsymbol{I}_P\right|^{\frac{\nu+I}{2}}} \\ =&\frac{\Gamma_P(\frac{\nu+I}{2})}{\Gamma_P(\frac{\nu}{2})} \frac{\pi^{-\frac{IP}{2}}}{\left|\sum_{i=1}^{I} \boldsymbol{\omega}_i \boldsymbol{\omega}_i^\top + \boldsymbol{I}_P\right|^{\frac{\nu+I}{2}}}. \end{aligned}$$

Finally, we can rewrite the sum in the denominator as a matrix product:

$$\frac{\Gamma_P(\frac{\nu+I}{2})}{\Gamma_P(\frac{\nu}{2})} \frac{\pi^{-\frac{IP}{2}}}{|\boldsymbol{\Omega}^\top \boldsymbol{\Omega} + \boldsymbol{I}_P|^{\frac{\nu+I}{2}}},$$

where $\boldsymbol{\Omega}$ is an $I \times P$ matrix which contains the P-dimensional random effects vectors $\boldsymbol{\omega}_i$ for all I participants (i.e., each row contains the random effects vector for one participant).

Appendix D: Rewriting the Warp-III Identity

Here we show how the expected value in the denominator of the bridge sampling identity when using Warp-III can be rewritten in such a way that we do not need to apply the warping transformation to the posterior samples that can range across the entire real line; instead, we can use those posterior samples directly. We start by inserting the warped posterior distribution in the bridge identity:

$$\Pr(\boldsymbol{N} = \boldsymbol{n}) = \frac{\displaystyle\int h(\boldsymbol{\eta})\, \tilde{p}_{\boldsymbol{\eta}}(\boldsymbol{\eta} \mid \boldsymbol{N} = \boldsymbol{n})\, g(\boldsymbol{\eta}) \mathrm{d}\boldsymbol{\eta}}{\displaystyle\int h(\boldsymbol{\eta})\, g(\boldsymbol{\eta})\, p_{\boldsymbol{\eta}}(\boldsymbol{\eta} \mid \boldsymbol{N} = \boldsymbol{n}) \mathrm{d}\boldsymbol{\eta}},$$

where

$$\begin{aligned} p_{\boldsymbol{\eta}}(\boldsymbol{\eta} \mid \boldsymbol{N} = \boldsymbol{n}) &= \frac{\frac{|\boldsymbol{R}|}{2} \left[\tilde{p}_{\boldsymbol{\psi}}(\boldsymbol{v} - \boldsymbol{R}\boldsymbol{\eta} \mid \boldsymbol{N} = \boldsymbol{n}) + \tilde{p}_{\boldsymbol{\psi}}(\boldsymbol{v} + \boldsymbol{R}\boldsymbol{\eta} \mid \boldsymbol{N} = \boldsymbol{n})\right]}{\Pr(\boldsymbol{N} = \boldsymbol{n})} \\ &= \frac{\tilde{p}_{\boldsymbol{\eta}}(\boldsymbol{\eta} \mid \boldsymbol{N} = \boldsymbol{n})}{\Pr(\boldsymbol{N} = \boldsymbol{n})}, \end{aligned}$$

and $g(\boldsymbol{\eta})$ denotes the multivariate standard normal proposal distribution. Next, we insert the expression for the "optimal" bridge function (note that the proportionality constant cancels since $h(\boldsymbol{\eta})$ appears both in the numerator and denominator):

$$\Pr(\boldsymbol{N} = \boldsymbol{n}) = \frac{\displaystyle\int \frac{\tilde{p}_{\boldsymbol{\eta}}(\boldsymbol{\eta}|\boldsymbol{N}=\boldsymbol{n})}{s_1\, \tilde{p}_{\boldsymbol{\eta}}(\boldsymbol{\eta}|\boldsymbol{N}=\boldsymbol{n}) + s_2 \Pr(\boldsymbol{N}=\boldsymbol{n})\, g(\boldsymbol{\eta})}\, g(\boldsymbol{\eta}) \mathrm{d}\boldsymbol{\eta}}{\displaystyle\int \frac{g(\boldsymbol{\eta})}{s_1\, \tilde{p}_{\boldsymbol{\eta}}(\boldsymbol{\eta}|\boldsymbol{N}=\boldsymbol{n}) + s_2 \Pr(\boldsymbol{N}=\boldsymbol{n})\, g(\boldsymbol{\eta})} p_{\boldsymbol{\eta}}(\boldsymbol{\eta} \mid \boldsymbol{N} = \boldsymbol{n}) \mathrm{d}\boldsymbol{\eta}}.$$

Now we multiply both the numerator and the denominator by $\frac{1/g(\boldsymbol{\eta})}{1/g(\boldsymbol{\eta})}$:

$$\Pr(\boldsymbol{N} = \boldsymbol{n}) = \frac{\displaystyle\int \frac{\frac{\tilde{p}_{\boldsymbol{\eta}}(\boldsymbol{\eta}|\boldsymbol{N}=\boldsymbol{n})}{g(\boldsymbol{\eta})}}{s_1 \frac{\tilde{p}_{\boldsymbol{\eta}}(\boldsymbol{\eta}|\boldsymbol{N}=\boldsymbol{n})}{g(\boldsymbol{\eta})} + s_2 \Pr(\boldsymbol{N}=\boldsymbol{n})}\, g(\boldsymbol{\eta}) \mathrm{d}\boldsymbol{\eta}}{\displaystyle\int \frac{1}{s_1 \frac{\tilde{p}_{\boldsymbol{\eta}}(\boldsymbol{\eta}|\boldsymbol{N}=\boldsymbol{n})}{g(\boldsymbol{\eta})} + s_2 \Pr(\boldsymbol{N}=\boldsymbol{n})} p_{\boldsymbol{\eta}}(\boldsymbol{\eta} \mid \boldsymbol{N} = \boldsymbol{n}) \mathrm{d}\boldsymbol{\eta}}.$$

The numerator looks good, since we can easily generate samples from g, the multivariate standard normal distribution. However, in the denominator, at the moment, we still have an expected value with respect to the warped posterior distribution. The goal is now to manipulate the denominator in a way that we obtain an expected value with respect to the posterior distribution of the parameters that can range across the entire real line but have not been warped (i.e., $p_{\boldsymbol{\psi}}(\boldsymbol{\psi} \mid \boldsymbol{N} = \boldsymbol{n})$). Hence, let us now focus on the denominator.

First, we insert the expression for the warped posterior:

$$\int \frac{1}{s_1 \frac{\frac{|\boldsymbol{R}|}{2}\left[\tilde{p}_{\boldsymbol{\psi}}(\boldsymbol{v}-\boldsymbol{R}\boldsymbol{\eta}|\boldsymbol{N}=\boldsymbol{n})+\tilde{p}_{\boldsymbol{\psi}}(\boldsymbol{v}+\boldsymbol{R}\boldsymbol{\eta}|\boldsymbol{N}=\boldsymbol{n})\right]}{g(\boldsymbol{\eta})} + s_2 \Pr(\boldsymbol{N}=\boldsymbol{n})} \times \frac{\frac{|\boldsymbol{R}|}{2}\left[\tilde{p}_{\boldsymbol{\psi}}(\boldsymbol{v}-\boldsymbol{R}\boldsymbol{\eta} \mid \boldsymbol{N}=\boldsymbol{n})+\tilde{p}_{\boldsymbol{\psi}}(\boldsymbol{v}+\boldsymbol{R}\boldsymbol{\eta} \mid \boldsymbol{N}=\boldsymbol{n})\right]}{\Pr(\boldsymbol{N}=\boldsymbol{n})} \mathrm{d}\boldsymbol{\eta}.$$

Second, we apply the following change-of-variable: $\boldsymbol{\psi} = \boldsymbol{R}\boldsymbol{\eta} + \boldsymbol{v}$ with $\mathrm{d}\boldsymbol{\eta} = |\boldsymbol{R}^{-1}|\mathrm{d}\boldsymbol{\psi}$. Hence, we obtain:

$$\int \frac{1}{s_1 \frac{\frac{|\boldsymbol{R}|}{2}\left[\tilde{p}_{\boldsymbol{\psi}}(2\boldsymbol{v}-\boldsymbol{\psi}|\boldsymbol{N}=\boldsymbol{n})+\tilde{p}_{\boldsymbol{\psi}}(\boldsymbol{\psi}|\boldsymbol{N}=\boldsymbol{n})\right]}{g(\boldsymbol{R}^{-1}(\boldsymbol{\psi}-\boldsymbol{v}))} + s_2 \Pr(\boldsymbol{N}=\boldsymbol{n})} \times \frac{\frac{1}{2}\left[\tilde{p}_{\boldsymbol{\psi}}(2\boldsymbol{v}-\boldsymbol{\psi} \mid \boldsymbol{N}=\boldsymbol{n})+\tilde{p}_{\boldsymbol{\psi}}(\boldsymbol{\psi} \mid \boldsymbol{N}=\boldsymbol{n})\right]}{\Pr(\boldsymbol{N}=\boldsymbol{n})} \mathrm{d}\boldsymbol{\psi}.$$

We can now split the integral into the sum of the following two integrals:

$$\frac{1}{2}\int \frac{1}{s_1 \frac{\frac{|\boldsymbol{R}|}{2}\left[\tilde{p}_{\boldsymbol{\psi}}(2\boldsymbol{v}-\boldsymbol{\psi}|\boldsymbol{N}=\boldsymbol{n})+\tilde{p}_{\boldsymbol{\psi}}(\boldsymbol{\psi}|\boldsymbol{N}=\boldsymbol{n})\right]}{g(\boldsymbol{R}^{-1}(\boldsymbol{\psi}-\boldsymbol{v}))} + s_2 \Pr(\boldsymbol{N}=\boldsymbol{n})} \frac{\tilde{p}_{\boldsymbol{\psi}}(2\boldsymbol{v}-\boldsymbol{\psi} \mid \boldsymbol{N}=\boldsymbol{n})}{\Pr(\boldsymbol{N}=\boldsymbol{n})} \mathrm{d}\boldsymbol{\psi}$$
$$+\frac{1}{2}\int \frac{1}{s_1 \frac{\frac{|\boldsymbol{R}|}{2}\left[\tilde{p}_{\boldsymbol{\psi}}(2\boldsymbol{v}-\boldsymbol{\psi}|\boldsymbol{N}=\boldsymbol{n})+\tilde{p}_{\boldsymbol{\psi}}(\boldsymbol{\psi}|\boldsymbol{N}=\boldsymbol{n})\right]}{g(\boldsymbol{R}^{-1}(\boldsymbol{\psi}-\boldsymbol{v}))} + s_2 \Pr(\boldsymbol{N}=\boldsymbol{n})} \frac{\tilde{p}_{\boldsymbol{\psi}}(\boldsymbol{\psi} \mid \boldsymbol{N}=\boldsymbol{n})}{\Pr(\boldsymbol{N}=\boldsymbol{n})} \mathrm{d}\boldsymbol{\psi}.$$

Next, we focus on the first integral in the sum and rewrite as:

$$\frac{1}{2}\int \frac{1}{s_1 \frac{\frac{|\boldsymbol{R}|}{2}\left[\tilde{p}_{\boldsymbol{\psi}}(-(\boldsymbol{\psi}-\boldsymbol{v})+\boldsymbol{v}|\boldsymbol{N}=\boldsymbol{n})+\tilde{p}_{\boldsymbol{\psi}}((\boldsymbol{\psi}-\boldsymbol{v})+\boldsymbol{v}|\boldsymbol{N}=\boldsymbol{n})\right]}{g(\boldsymbol{R}^{-1}(\boldsymbol{\psi}-\boldsymbol{v}))} + s_2 \Pr(\boldsymbol{N}=\boldsymbol{n})} \frac{\tilde{p}_{\boldsymbol{\psi}}(-(\boldsymbol{\psi}-\boldsymbol{v})+\boldsymbol{v} \mid \boldsymbol{N}=\boldsymbol{n})}{\Pr(\boldsymbol{N}=\boldsymbol{n})} \mathrm{d}\boldsymbol{\psi}.$$

The trick is now to take the "mirror image" around $\boldsymbol{v}$ which will not change the value of the integral but is convenient for what follows (see also Ardia, Baştürk, Hoogerheide, & van Dijk, 2012). The easiest way of understanding why taking the mirror image around $\boldsymbol{v}$ does not change the value of the integral is to consider the one-dimensional case. In this case, the value of the integral corresponds to an area. Taking the mirror image around an axis parallel to the y-axis with location determined by the mirror point has the consequence that the area that has been to the right of that mirror point is now to the left of that mirror point and vice-versa, however, the total area (i.e., the value of the integral) remains the same (remember that we are integrating across the entire real line). We can then rewrite the integral as:

$$
\begin{aligned}
&\frac{1}{2}\int \frac{1}{s_1 \frac{\frac{|\boldsymbol{R}|}{2}\left[\tilde{p}_{\boldsymbol{\psi}}((\boldsymbol{\psi}-\boldsymbol{v})+\boldsymbol{v}|\boldsymbol{N}=\boldsymbol{n})+\tilde{p}_{\boldsymbol{\psi}}(-(\boldsymbol{\psi}-\boldsymbol{v})+\boldsymbol{v}|\boldsymbol{N}=\boldsymbol{n})\right]}{g(\boldsymbol{R}^{-1}(\boldsymbol{\psi}-\boldsymbol{v}))} + s_2 \Pr(\boldsymbol{N}=\boldsymbol{n})} \frac{\tilde{p}_{\boldsymbol{\psi}}((\boldsymbol{\psi}-\boldsymbol{v})+\boldsymbol{v} \mid \boldsymbol{N}=\boldsymbol{n})}{\Pr(\boldsymbol{N}=\boldsymbol{n})} \mathrm{d}\boldsymbol{\psi} \\
=&\frac{1}{2}\int \frac{1}{s_1 \frac{\frac{|\boldsymbol{R}|}{2}\left[\tilde{p}_{\boldsymbol{\psi}}(2\boldsymbol{v}-\boldsymbol{\psi}|\boldsymbol{N}=\boldsymbol{n})+\tilde{p}_{\boldsymbol{\psi}}(\boldsymbol{\psi}|\boldsymbol{N}=\boldsymbol{n})\right]}{g(\boldsymbol{R}^{-1}(\boldsymbol{\psi}-\boldsymbol{v}))} + s_2 \Pr(\boldsymbol{N}=\boldsymbol{n})} \frac{\tilde{p}_{\boldsymbol{\psi}}(\boldsymbol{\psi} \mid \boldsymbol{N}=\boldsymbol{n})}{\Pr(\boldsymbol{N}=\boldsymbol{n})} \mathrm{d}\boldsymbol{\psi},
\end{aligned}
$$

where we made use of the fact that g is symmetric with respect to the origin. We notice that the integral now looks the same as the second integral in the sum of the two integrals that we considered before. Hence, we can combine the two and obtain the following for the

denominator of the bridge identity:

$$\int \frac{1}{s_1 \frac{\frac{|\boldsymbol{R}|}{2}\left[\tilde{p}_{\boldsymbol{\psi}}(2\boldsymbol{v}-\boldsymbol{\psi}|\boldsymbol{N}=\boldsymbol{n})+\tilde{p}_{\boldsymbol{\psi}}(\boldsymbol{\psi}|\boldsymbol{N}=\boldsymbol{n})\right]}{g(\boldsymbol{R}^{-1}(\boldsymbol{\psi}-\boldsymbol{v}))} + s_2 \Pr(\boldsymbol{N}=\boldsymbol{n})} \frac{\tilde{p}_{\boldsymbol{\psi}}(\boldsymbol{\psi} \mid \boldsymbol{N}=\boldsymbol{n})}{\Pr(\boldsymbol{N}=\boldsymbol{n})} \mathrm{d}\boldsymbol{\psi}$$
$$= \int \frac{1}{s_1 \frac{\frac{|\boldsymbol{R}|}{2}\left[\tilde{p}_{\boldsymbol{\psi}}(2\boldsymbol{v}-\boldsymbol{\psi}|\boldsymbol{N}=\boldsymbol{n})+\tilde{p}_{\boldsymbol{\psi}}(\boldsymbol{\psi}|\boldsymbol{N}=\boldsymbol{n})\right]}{g(\boldsymbol{R}^{-1}(\boldsymbol{\psi}-\boldsymbol{v}))} + s_2 \Pr(\boldsymbol{N}=\boldsymbol{n})} p_{\boldsymbol{\psi}}(\boldsymbol{\psi} \mid \boldsymbol{N}=\boldsymbol{n}) \mathrm{d}\boldsymbol{\psi}.$$

This can be interpreted as an expected value with respect to the posterior samples that have been transformed to the real line but have not been warped. Using this expression in the bridge identity, we obtain:

$$\Pr(\boldsymbol{N}=\boldsymbol{n}) = \frac{\int \frac{\frac{\tilde{p}_{\boldsymbol{\eta}}(\boldsymbol{\eta}|\boldsymbol{N}=\boldsymbol{n})}{g(\boldsymbol{\eta})}}{s_1 \frac{\tilde{p}_{\boldsymbol{\eta}}(\boldsymbol{\eta}|\boldsymbol{N}=\boldsymbol{n})}{g(\boldsymbol{\eta})} + s_2 \Pr(\boldsymbol{N}=\boldsymbol{n})} g(\boldsymbol{\eta}) \mathrm{d}\boldsymbol{\eta}}{\int \frac{1}{s_1 \frac{\frac{|\boldsymbol{R}|}{2}\left[\tilde{p}_{\boldsymbol{\psi}}(2\boldsymbol{v}-\boldsymbol{\psi}|\boldsymbol{N}=\boldsymbol{n})+\tilde{p}_{\boldsymbol{\psi}}(\boldsymbol{\psi}|\boldsymbol{N}=\boldsymbol{n})\right]}{g(\boldsymbol{R}^{-1}(\boldsymbol{\psi}-\boldsymbol{v}))} + s_2 \Pr(\boldsymbol{N}=\boldsymbol{n})} p_{\boldsymbol{\psi}}(\boldsymbol{\psi} \mid \boldsymbol{N}=\boldsymbol{n}) \mathrm{d}\boldsymbol{\psi}}$$
$$= \frac{\mathbb{E}_{g(\boldsymbol{\eta})}\left[\frac{\frac{\frac{|\boldsymbol{R}|}{2}\left[\tilde{p}_{\boldsymbol{\psi}}(\boldsymbol{v}-\boldsymbol{R}\boldsymbol{\eta}|\boldsymbol{N}=\boldsymbol{n})+\tilde{p}_{\boldsymbol{\psi}}(\boldsymbol{v}+\boldsymbol{R}\boldsymbol{\eta}|\boldsymbol{N}=\boldsymbol{n})\right]}{g(\boldsymbol{\eta})}}{s_1 \frac{\frac{|\boldsymbol{R}|}{2}\left[\tilde{p}_{\boldsymbol{\psi}}(\boldsymbol{v}-\boldsymbol{R}\boldsymbol{\eta}|\boldsymbol{N}=\boldsymbol{n})+\tilde{p}_{\boldsymbol{\psi}}(\boldsymbol{v}+\boldsymbol{R}\boldsymbol{\eta}|\boldsymbol{N}=\boldsymbol{n})\right]}{g(\boldsymbol{\eta})} + s_2 \Pr(\boldsymbol{N}=\boldsymbol{n})}\right]}{\mathbb{E}_{p_{\boldsymbol{\psi}}(\boldsymbol{\psi}|\boldsymbol{N}=\boldsymbol{n})}\left[\frac{1}{s_1 \frac{\frac{|\boldsymbol{R}|}{2}\left[\tilde{p}_{\boldsymbol{\psi}}(2\boldsymbol{v}-\boldsymbol{\psi}|\boldsymbol{N}=\boldsymbol{n})+\tilde{p}_{\boldsymbol{\psi}}(\boldsymbol{\psi}|\boldsymbol{N}=\boldsymbol{n})\right]}{g(\boldsymbol{R}^{-1}(\boldsymbol{\psi}-\boldsymbol{v}))} + s_2 \Pr(\boldsymbol{N}=\boldsymbol{n})}\right]}.$$

These two expected values are then estimated using the iterative updating scheme as follows:

$$\hat{\Pr}(\boldsymbol{N}=\boldsymbol{n})^{(t+1)} = \frac{\frac{1}{D_2} \sum_{r=1}^{D_2} \frac{l_{2,r}}{s_1 \, l_{2,r} + s_2 \, \hat{\Pr}(\boldsymbol{N}=\boldsymbol{n})^{(t)}}}{\frac{1}{D_1} \sum_{j=1}^{D_1} \frac{1}{s_1 \, l_{1,j} + s_2 \, \hat{\Pr}(\boldsymbol{N}=\boldsymbol{n})^{(t)}}}, \tag{2}$$

where

$$l_{1,j} = \frac{\frac{|\boldsymbol{R}|}{2}\left[\tilde{p}_{\boldsymbol{\psi}}(2\boldsymbol{v} - \boldsymbol{\psi}_j^* \mid \boldsymbol{N} = \boldsymbol{n}) + \tilde{p}_{\boldsymbol{\psi}}(\boldsymbol{\psi}_j^* \mid \boldsymbol{N} = \boldsymbol{n})\right]}{g\left(\boldsymbol{R}^{-1}\left(\boldsymbol{\psi}_j^* - \boldsymbol{v}\right)\right)}, \tag{3}$$

and

$$l_{2,r} = \frac{\frac{|\boldsymbol{R}|}{2}\left[\tilde{p}_{\boldsymbol{\psi}}(\boldsymbol{v} - \boldsymbol{R}\tilde{\boldsymbol{\eta}}_r \mid \boldsymbol{N} = \boldsymbol{n}) + \tilde{p}_{\boldsymbol{\psi}}(\boldsymbol{v} + \boldsymbol{R}\tilde{\boldsymbol{\eta}}_r \mid \boldsymbol{N} = \boldsymbol{n})\right]}{g(\tilde{\boldsymbol{\eta}}_r)}. \tag{4}$$

$\{\boldsymbol{\psi}_1^*, \ldots, \boldsymbol{\psi}_{D_1}^*\}$ are D_1 draws from the posterior distribution of the parameters that can range across the entire real line $p_{\boldsymbol{\psi}}(\boldsymbol{\psi} \mid \boldsymbol{N} = \boldsymbol{n})$, and $\{\tilde{\boldsymbol{\eta}}_1, \ldots, \tilde{\boldsymbol{\eta}}_{D_2}\}$ are D_2 draws from the multivariate standard normal distribution $g(\boldsymbol{\eta})$.

Appendix E: Additional Information Example 1

Details about Priors for $\boldsymbol{\delta}$

Here we explain how the standard deviations of the zero-centered normal prior distributions for the components of the trial effects vector $\boldsymbol{\delta}$ were chosen. To explain how these prior standard deviations were chosen, we focus on one MPT parameter, c. When μ_c is set to its prior mean zero, the probit group mean for trial one is equal to $-\delta_c/2$ and for trial two, it is equal to $\delta_c/2$. Since we use normal prior distributions with mean zero for δ_c, we know that about 95% of the prior mass falls within +/- 2 standard deviations. Hence, the trial difference that is obtained when using the δ_c that corresponds to two prior standard deviations can be considered approximately the largest expected trial difference under this prior choice. To choose the prior standard deviation, we reverted this procedure: (1) we specified the largest expected trial difference on the probability scale symmetric around

0.5; (2) we used these values, inserted two times the prior standard deviation for δ_c in the equation for the trial means on the probability scale, and then solved for the prior standard deviation which we call σ_δ. We explored three different choices for the largest expected trial difference which yielded three different σ_δ. For the first prior (narrow prior), we set the value that corresponds to about the largest expected trial difference on the probability scale equal to 0.4 (i.e., the trial values were 0.3 and 0.7). The prior standard deviation $\sigma_\delta^{\text{narrow}}$ was then obtained as follows:

$$\Phi^{-1}(0.3) = -\frac{2\sigma_\delta^{\text{narrow}}}{2} \text{ and } \Phi^{-1}(0.7) = \frac{2\sigma_\delta^{\text{narrow}}}{2}. \tag{5}$$

Hence,

$$\begin{gathered} \Phi^{-1}(0.3) = -\sigma_\delta^{\text{narrow}} \text{ and } \Phi^{-1}(0.7) = \sigma_\delta^{\text{narrow}} \\ \sigma_\delta^{\text{narrow}} \approx 0.52. \end{gathered} \tag{6}$$

For the medium prior, we set the largest expected trial difference on the probability scale equal to 0.6 (i.e., the trial values were 0.2 and 0.8) and repeated the above described procedure: this yielded $\sigma_\delta^{\text{medium}} \approx 0.84$. For the wide prior, the largest expected trial difference on the probability scale was set to 0.8 (i.e., the trial values were 0.1 and 0.9): this yielded $\sigma_\delta^{\text{wide}} \approx 1.28$.

Results for Alternative Prior $\xi_{\textbf{max}} = 2$

Here we present the results for example 1 that are obtained when using the alternative uniform prior with upper bound $\xi_{\text{max}} = 2$ instead of $\xi_{\text{max}} = 10$ on the components of $\boldsymbol{\xi}$ that has been chosen based on the pattern of implied group-level distributions on the probability scale (described above). Figure 3 displays the posterior distributions of the to the probability scale transformed probit parameter means for trial one and two for the full model and Figure 4 shows the posterior model probabilities (left panel) and the posterior inclusion probabilities (right panel). The figures highlight that the results are highly similar to the ones that are obtained when using uniform priors with upper bound $\xi_{\text{max}} = 10$ on the components of $\boldsymbol{\xi}$.

Implementation Check via Savage-Dickey Density Ratio

To check that we implemented the Warp-III procedure correctly, we exploited the fact that for the nested model comparisons of example 1, we can also obtain the relevant quantities via the Savage-Dickey density ratio representation of the Bayes factor (Dickey & Lientz, 1970). The Savage-Dickey density representation uses the fact that the Bayes factor that compares a model which allows a parameter to be freely estimated compared to a nested model which fixes this parameter (e.g., to zero) is given by the ratio of the prior to the posterior density of that parameter under the more complex model evaluated at the test value (for a tutorial and an explanation under which circumstances this trick can be applied see Wagenmakers, Lodewyckx, Kuriyal, & Grasman, 2010). Hence, we first computed a

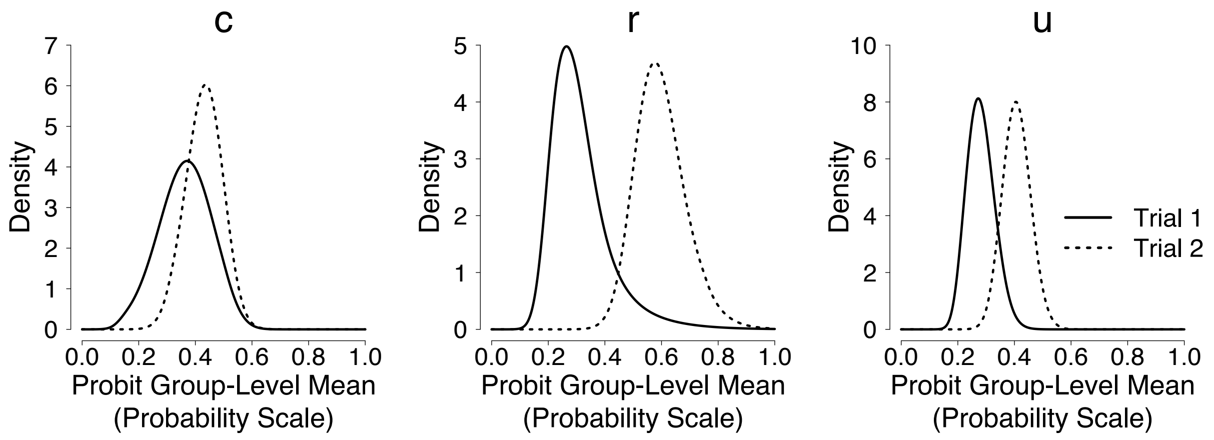

Figure 3: Posterior distributions of the probit group-level means (plotted on the probability scale) from the full model $\mathcal{M}_1$ for the analysis of the first two trials of the pair-clustering data reported in Riefer et al. (2002) based on the prior choice $\xi_{\text{max}} = 2$. The solid lines correspond to the posteriors for the first trial, the dotted lines to the posteriors for the second trial. The results are almost identical to the ones based on $\xi_{\text{max}} = 10$. Figure available at `https://tinyurl.com/y8vr2ugt` under CC license `https://creativecommons.org/licenses/by/2.0/`.

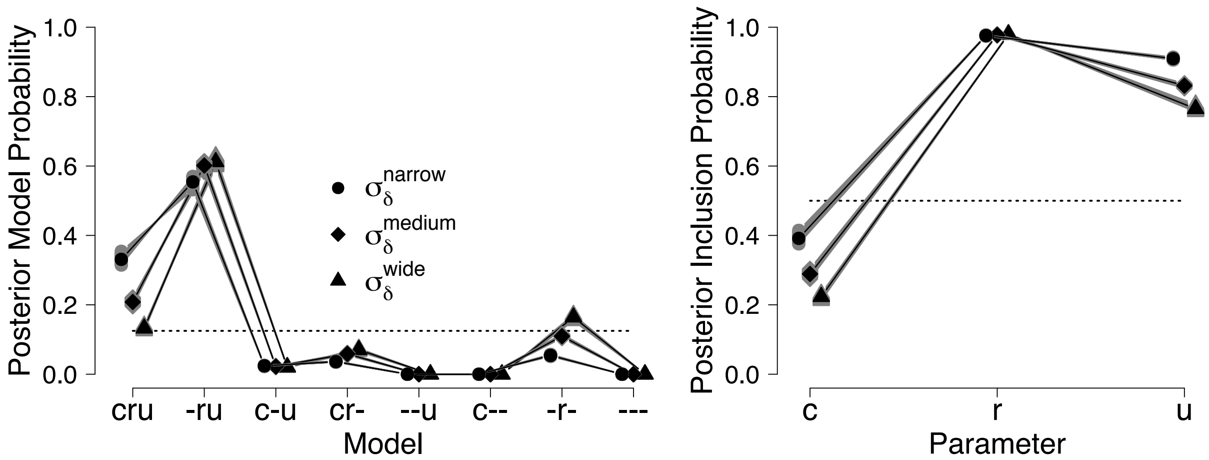

Figure 4: Posterior model probabilities (left panel) and posterior inclusion probabilities (right panel) for the analysis of the first two trials of the pair-clustering data reported in Riefer et al. (2002) obtained with Warp-III bridge sampling based on the prior choice $\xi_{\max} = 2$. In the left panel, the x-axis indicates which parameters were allowed to vary from the first to the second trial (e.g., $c-u$ corresponds to $\mathcal{M}_3$ where r was fixed between trials). Gray symbols show the results of the 50 repetitions and black symbols display the posterior model probabilities and posterior inclusion probabilities that are based on the median of the 50 estimated log marginal likelihoods. Circles show results obtained with the narrow prior, diamonds with the medium prior, and triangles with the wide prior. The dotted lines show the prior model probabilities and prior inclusion probabilities. The results are almost identical to the ones based on $\xi_{\max} = 10$. Available at `https://tinyurl.com/y7yazlk2` under CC license `https://creativecommons.org/licenses/by/2.0/`.

set of Bayes factors via the Savage-Dickey approach and then converted them to posterior model probabilities which were then compared to the posterior model probabilities obtained via Warp-III. The same comparison was conducted for the posterior inclusion probabilities. The results of these comparisons are shown in Figure 5 (based on $\xi_{\text{max}} = 10$). As in the main text, the results of the 50 Warp-III repetitions are depicted as gray symbols. However, in contrast to the figure in the main text, the black symbols do not correspond to the results based on the median of the estimated log marginal likelihoods but correspond to the results based on the Savage-Dickey density ratio approach. Figure 5 shows that the results of Warp-III and the Savage-Dickey density ratio approach are highly similar and thus confirms a successful implementation of the Warp-III procedure.

Appendix F: Additional Information Example 2

Posterior Distributions

Here we present the posterior distributions of the probit mean parameters plotted on the probability scale for the non-nested example from Fazio, Brashier, Payne, and Marsh (2015). Figure 6 shows the posterior distributions based on uniform priors with upper bound $\xi_{\text{max}} = 10$ for the components of $\boldsymbol{\xi}$. The results for uniform priors with upper bound $\xi_{\text{max}} = 2$ were highly similar and are not displayed.

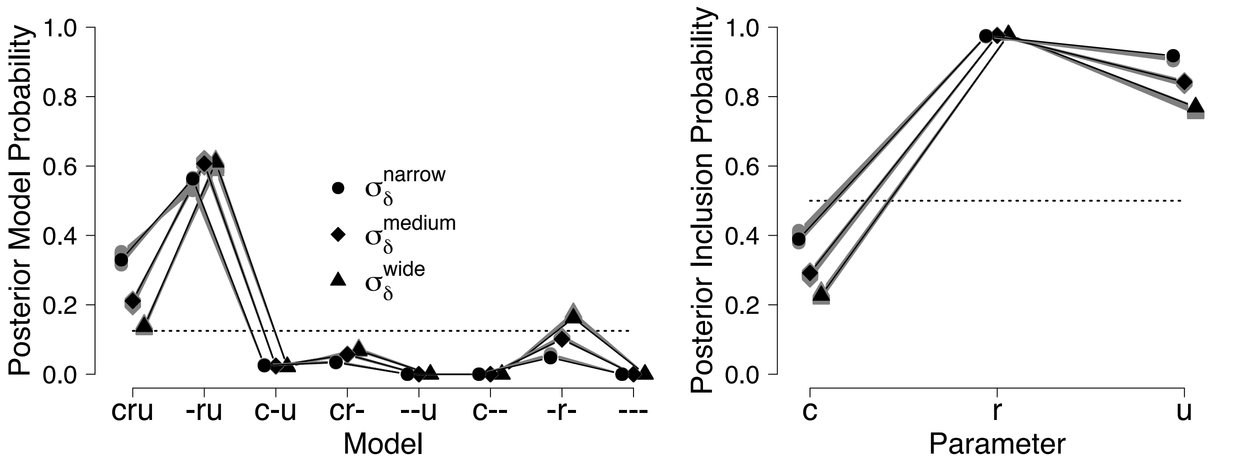

Figure 5: Posterior model probabilities (left panel) and posterior inclusion probabilities (right panel) obtained with Warp-III and the Savage-Dickey density ratio approach. In the left panel, the x-axis indicates which parameters were allowed to vary from the first to the second trial (e.g., $c - u$ corresponds to $\mathcal{M}_3$ where r was fixed between trials). Gray symbols show the results of the 50 repetitions and black symbols display the posterior model probabilities and posterior inclusion probabilities that are based on the Savage-Dickey density ratio approach. Circles show results obtained with the narrow prior, diamonds with the medium prior, and triangles with the wide prior. The dotted lines show the prior model probabilities and prior inclusion probabilities. The results based on Warp-III are very similar to the ones based on the Savage-Dickey density ratio approach which confirms a successful implementation of Warp-III. Available at `https://tinyurl.com/ycsyqlpu` under CC license `https://creativecommons.org/licenses/by/2.0/`.

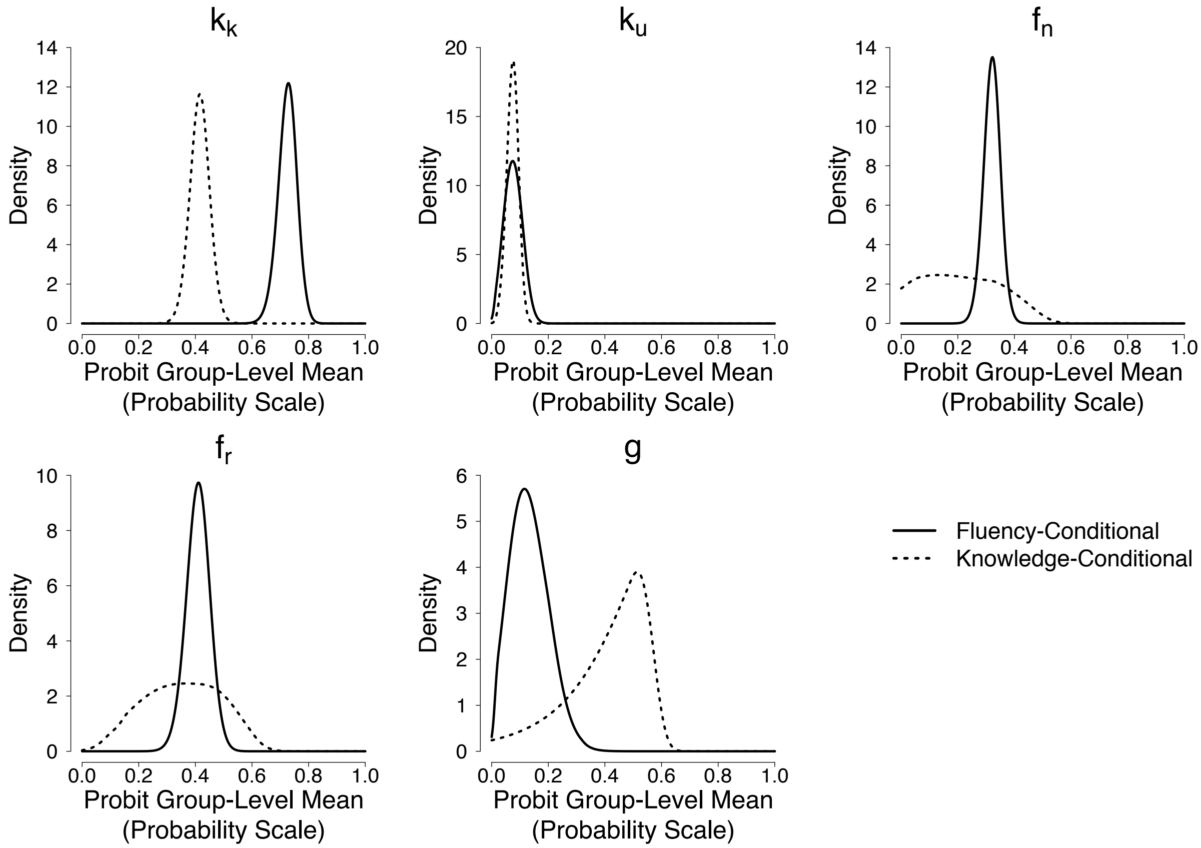

Figure 6: Posterior distributions of the probit group-level means plotted on the probability scale for the non-nested example from Fazio et al. (2015). The solid lines correspond to the posterior distributions for the fluency-conditional model, the dotted lines correspond to the posterior distributions for the knowledge-conditional model. Figure available at `https://tinyurl.com/ya6y8bvc` under CC license `https://creativecommons.org/licenses/by/2.0/`.

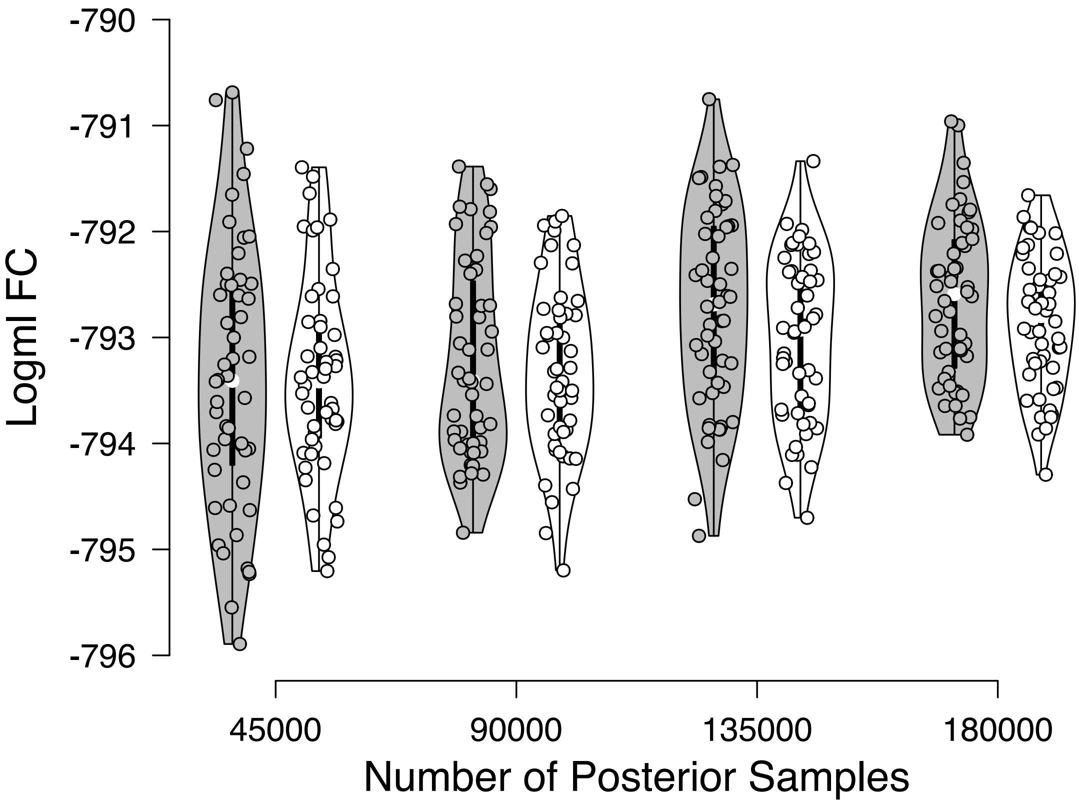

Figure 7: Log marginal likelihood estimates for the fluency-conditional (FC) model as a function of the number of posterior samples. The Warp-III estimates are displayed in white, the estimates based on the simpler multivariate normal approach are displayed in gray. Available at `https://tinyurl.com/y8ku5gln` under CC license `https://creativecommons.org/licenses/by/2.0/`.

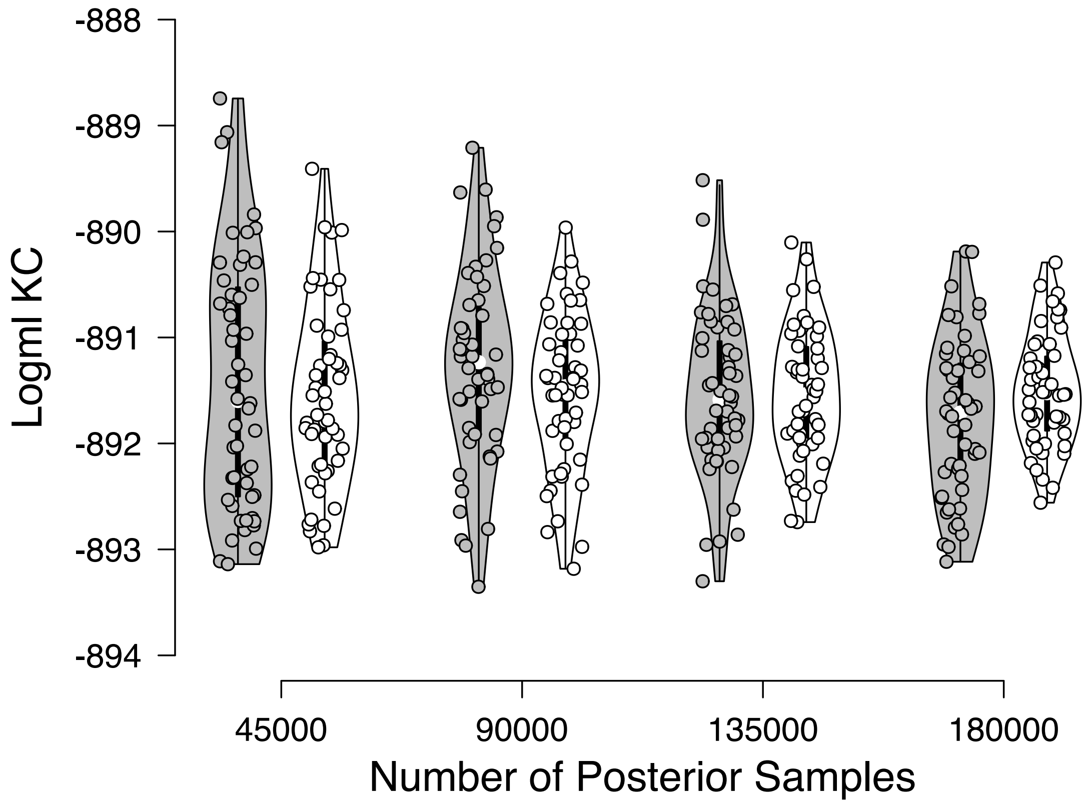

Figure 8: Log marginal likelihood estimates for the knowledge-conditional (KC) model as a function of the number of posterior samples. The Warp-III estimates are displayed in white, the estimates based on the simpler multivariate normal approach are displayed in gray. Available at `https://tinyurl.com/ybo3fys9` under CC license `https://creativecommons.org/licenses/by/2.0/`.

Log Marginal Likelihood Estimates

Here we present plots of the log marginal likelihood estimates based on Warp-III and based on the simpler multivariate normal bridge sampling approach as a function of the number of posterior samples. Figure 7 displays the log marginal likelihood estimates for the fluency-conditional model, Figure 8 displays the results for the knowledge-conditional model. For this particular example it is apparent that the Warp-III estimates are less variable than the estimates based on the simpler multivariate normal approach.

References

Ardia, D., Baştürk, N., Hoogerheide, L., & van Dijk, H. K. (2012). A comparative study of Monte Carlo methods for efficient evaluation of marginal likelihood. *Computational Statistics and Data Analysis*, *56*, 3398–3414.

Dickey, J. M., & Lientz, B. P. (1970). The weighted likelihood ratio, sharp hypotheses about chances, the order of a Markov chain. *The Annals of Mathematical Statistics*, *41*, 214–226.

Fazio, L. K., Brashier, N. M., Payne, B. K., & Marsh, E. J. (2015). Knowledge does not protect against illusory truth. *Journal of Experimental Psychology: General*, *144*, 993–1002.

Heck, D. W., Arnold, N. R., & Arnold, D. (2018). TreeBUGS: An R package for hierarchical multinomial-processing-tree modeling. *Behavior Research Methods*, *50*(1), 264–284.

Klauer, K. C. (2010). Hierarchical multinomial processing tree models: A latent–trait approach. *Psychometrika*, *75*, 70–98.

Matzke, D., Dolan, C. V., Batchelder, W. H., & Wagenmakers, E.-J. (2015). Bayesian estimation of multinomial processing tree models with heterogeneity in participants and items. *Psychometrika*, *80*, 205–235.

Riefer, D. M., Knapp, B. R., Batchelder, W. H., Bamber, D., & Manifold, V. (2002). Cognitive psychometrics: Assessing storage and retrieval deficits in special populations with multinomial processing tree models. *Psychological Assessment*, *14*, 184–201.

Wagenmakers, E.-J., Lodewyckx, T., Kuriyal, H., & Grasman, R. (2010). Bayesian hypothesis testing for psychologists: A tutorial on the Savage–Dickey method. *Cognitive Psychology*, *60*, 158–189.
